# Liraglutide restores impaired associative learning in individuals with obesity

**DOI:** 10.1038/s42255-023-00859-y

**Published:** 2023-08-17

**Authors:** Ruth Hanssen, Lionel Rigoux, Bojana Kuzmanovic, Sandra Iglesias, Alina C. Kretschmer, Marc Schlamann, Kerstin Albus, Sharmili Edwin Thanarajah, Tamara Sitnikow, Corina Melzer, Oliver A. Cornely, Jens C. Brüning, Marc Tittgemeyer

**Affiliations:** 1 Max Planck Institute for Metabolism Research, Cologne, Germany; 2 Faculty of Medicine and University Hospital Cologne, Policlinic for Endocrinology, Diabetology and Preventive Medicine (PEPD), University of Cologne, Cologne, Germany; 3 Translational Neuromodeling Unit, Institute for Biomedical Engineering, University of Zurich and Swiss Federal Institute of Technology, Zurich, Switzerland; 4 Faculty of Medicine and University Hospital Cologne, Department I of Internal Medicine, Center for Integrated Oncology Aachen Bonn Cologne Duesseldorf (CIO ABCD) and Excellence Center for Medical Mycology (ECMM), University of Cologne, Cologne, Germany; 5 Faculty of Medicine and University Hospital Cologne, Institute for Diagnostic and Interventional Radiology, University of Cologne, Cologne, Germany; 6 Cologne Excellence Cluster on Cellular Stress Responses in Aging-Associated Diseases (CECAD), University of Cologne, Cologne, Germany; 7 Department of Psychiatry, Psychosomatic Medicine and Psychotherapy, University Hospital Frankfurt, Frankfurt am Main, Germany; 8 German Centre for Infection Research (DZIF), Partner Site Bonn-Cologne, Cologne, Germany; 9 Faculty of Medicine and University Hospital Cologne, Clinical Trials Centre Cologne (ZKS Köln), University of Cologne, Cologne, Germany

**Keywords:** Metabolism, Learning algorithms, Endocrine system and metabolic diseases

## Abstract

Survival under selective pressure is driven by the ability of our brain to use sensory information to our advantage to control physiological needs. To that end, neural circuits receive and integrate external environmental cues and internal metabolic signals to form learned sensory associations, consequently motivating and adapting our behaviour. The dopaminergic midbrain plays a crucial role in learning adaptive behaviour and is particularly sensitive to peripheral metabolic signals, including intestinal peptides, such as glucagon-like peptide 1 (GLP-1). In a single-blinded, randomized, controlled, crossover basic human functional magnetic resonance imaging study relying on a computational model of the adaptive learning process underlying behavioural responses, we show that adaptive learning is reduced when metabolic sensing is impaired in obesity, as indexed by reduced insulin sensitivity (participants: *N* = 30 with normal insulin sensitivity; *N* = 24 with impaired insulin sensitivity). Treatment with the GLP-1 receptor agonist liraglutide normalizes impaired learning of sensory associations in men and women with obesity. Collectively, our findings reveal that GLP-1 receptor activation modulates associative learning in people with obesity via its central effects within the mesoaccumbens pathway. These findings provide evidence for how metabolic signals can act as neuromodulators to adapt our behaviour to our body’s internal state and how GLP-1 receptor agonists work in clinics.

## Abstract (web summary)

Hanssen et al. demonstrate that associative learning is reduced when metabolic sensing is impaired in obesity and can be restored by liraglutide.

## Main

Learning associations emerging from the sensory information that we perceive in a changing environment are essential to survive and thrive under selective pressure^1^. Through associative learning, sensory signals gain a motivational force and enable our brains to direct our actions and hence adapt our behaviour to maintain an organism’s fitness. Associative learning (whose evolutionary origin has long been considered fundamental to behavioural adaptation^2^) is historically presumed to rely foremost on information provided by the body’s sensory systems about the external environment, which the brain must interpret to select a behavioural response^3^. However, human and non-human behaviour is highly malleable and adapts successfully not only to external constraints but also to internal demands^4^.

The regulation of energy balance, for instance, requires our behaviour to adapt to our physiological needs^5,6^. Hence, our brain has to receive, integrate and prioritize physiological signals conveying information about the homeostatic state^7,8^. To this end, metabolically relevant signals reflecting physiological needs are communicated through parallel pathways^9^ from the periphery to the brain. These signals are eventually processed with sensory cues from the external environment to drive motivated behaviour and prompt food intake^10,11^. More precisely, metabolic sensing of homeostatic state can modulate the value of stimuli and actions^12,13^, thus promoting motivated behavioural responses^14^ and inducing learning of new outcome associations^15^ involved in the rapid detection of physiologically relevant sensory cues (from the body and the external environment)^16^.

On a neural level, dopamine (DA) neurons of the ventral midbrain and their projection targets promote adaptive behaviour by regulating motivation and reinforcing actions through DA-dependent plasticity^17–19^. Specifically, the mesoaccumbens pathway, that is, the DA projection from the ventral tegmental area (VTA) to the nucleus accumbens (NAc), is critical for learning from rewards^20,21^. Indeed, VTA dopaminergic neurons encode so-called reward prediction errors, vital learning signals in computational theories to formalize the neurobiological implementation of motivated behaviour in algorithms for reconstructing a reward distribution from experience^22^. Reward prediction errors are defined as the mismatch between the actual and expected values of the outcomes of an action^23^. These errors effectively allow us to update our predictions about which outcomes are likely to be beneficial in a particular context and thus dynamically direct our choices toward optimal behaviours^24^.

Furthermore, the amplitude of an error needs to be put in perspective with the precision of the relative prediction^25^. Higher-order statistical properties of the learned associations, such as the variance of the outcome or volatility of its expectation, should down- or upweight the prediction error’s influence to optimize learning^26^. Such adaptive encoding of prediction errors has been demonstrated in the neural response of the mesoaccumbens DA pathway in monkeys and humans^27,28^, matching theoretical models of adaptive learning.

In the more general context of associative learning, and in addition to the above, prediction errors indicate a need to update current beliefs about incoming sensory inputs^26,29,30^. However, the relevance of these inputs needs to be evaluated in light of the current physiological status to support a need-appropriate outcome evaluation and adaptively guide choice behaviour^31,32^. The mesoaccumbens pathway recently emerged as a strong candidate for this contextualization of the learning process to metabolic sensing of homeostatic state; related to food intake, VTA DA neurons are susceptible to the nutritional value of food cues^33,34^ and postingestive effects of food^35,36^ and are also strongly modulated by peripheral orexigenic and anorexigenic peptides^37–39^. Insulin and glucagon-like peptide 1 (GLP-1) receptors are particularly prominent examples^40–42^. Both corresponding circulating peptides affect feeding and downregulate DA activity^43–45^. While the impact of circulating insulin on food intake is controversial, specifically, activation of GLP-1 receptors in the VTA by endogenous GLP-1 reduces the excitatory synaptic strength of VTA DA neurons projecting to the NAc^46^.

Similarly, insulin action on these neurons depresses excitatory synaptic transmission^44^, decreases DA concentrations by enhancing its clearance^45,47^ and reduces DA release into the NAc^43^. In effect, insulin can reduce anticipatory activity and the formation of preference for food-related cues^44^. Both insulin and GLP-1 affect motivation to work for reward in rodents^40^ and in humans^48^. However, while such modulation of DA neurons also predicts that metabolic signals should alter prediction error encoding, direct evidence for the role of GLP-1 and insulin in the regulation of associative learning is still lacking.

In line with this hypothesis, the overconsumption of food and, ultimately, obesity relate to metabolic impairments reflected by reduced insulin sensitivity^49,50^, possibly insufficient GLP-1 signalling^51–53^, notable alterations in mesoaccumbens DA function^34,54^ and impaired outcome learning^55–57^. Together, these observations suggest that a lack of integration of peripheral metabolic signals into DA function could contribute to maladaptive behaviour, as seen in obesity, particularly by disrupting the sensitivity of learning mechanisms to physiological needs.

Intriguingly, recent evidence suggests that GLP-1 receptor agonists augment glucose-dependent insulin release^51^ and can restore motivational behaviour in insulin-resistant humans^48^. We therefore hypothesized that altered metabolic functioning indexed by impaired insulin sensitivity would impair the learning of sensory associations and that augmentation of dysregulated metabolic functioning with a GLP-1 receptor agonist^41^ might alleviate this impairment.

Therefore, we performed a randomized, placebo-controlled crossover functional magnetic resonance imaging (fMRI) study to assess the effect of GLP-1 receptor activation on associative learning in humans with and without metabolic dysfunction. We recruited as participants lean individuals and those with obesity and assessed their peripheral insulin sensitivity using the homeostasis model assessment of insulin resistance (HOMA-IR)^58^ as a proxy for whole-body insulin sensitivity. Each participant completed a sensory associative learning task^59^ during fMRI on two separate days, either under placebo conditions or under intervention with the GLP-1 analogue liraglutide. We used a computational model of adaptive sensory learning already proven with this task to provide reliable estimates of individual learning performance^60^ and to reveal adaptive prediction error encoding in the ventral striatum and midbrain^59^. Thus, here, we strive to investigate the role of metabolic impairment and the potency of a GLP-1 receptor agonist to affect DA-driven prediction error signalling. We demonstrate that DA-driven prediction error learning of external sensory cues critically depends on metabolic signalling.

## Results

**Fig. 1:**
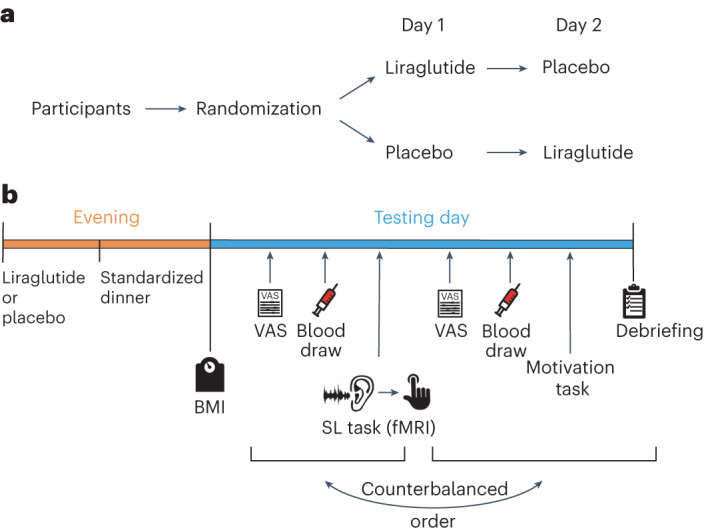
Experimental design. **a**, Randomization of participants. In this interventional crossover study, all participants underwent a placebo and a liraglutide session. The order of the interventions was counterbalanced across participants. **b**, Outline of the testing day; BMI, body mass index; VAS, visual analogue scale used for hunger rating; SL task, associative sensory learning task assessing trial-wise learning processes.

We report findings from human participants with normal (IS^+^ group) or impaired (IS^–^ group) peripheral insulin sensitivity who performed a computerized associative learning task while undergoing fMRI on two separate days. Participants received the GLP-1 analogue liraglutide on the first day and placebo on the second day (Fig. 1a). The task assessed the ability of participants to learn associations between auditory cues (a high or low tone) and a subsequent visual outcome (a picture of a face or a house). During the experiment, these associations fluctuated between being highly predictable (for example, a high tone predicts a face with a probability of 0.9) and unpredictable (that is, both tones predict a face with an equal probability of 0.5), generating varying volatilities and requiring adaptive learning. Learning about the predictive power of the auditory stimuli was modelled by a hierarchical Bayesian updating process as implemented in the hierarchical Gaussian filter (HGF^61^; Methods). Unlike alternative models of learning assuming a fixed learning rate, the HGF allows for an online adaptation of the learning rate^60^ and thus better reflects the adaptive prediction error encoding seen in DA neurons^28^.

### Behavioural analysis

We first fitted the HGF model to the sequence of choices from each participant for each session to estimate the individual trajectories of cue–outcome association learning. Thus, we recovered three critical covert computational quantities reflecting the learning process: the sensory prediction error, the adaptive learning rate and, as a product, the adaptive prediction error. The sensory prediction error constitutes the discrepancy between the correctness of the participant’s choice and the expectation of this choice being correct. The learning rate weights the sensory prediction error by considering the current subjective uncertainty about the cue–outcome association and thereby modulates its impact on learning; note the learning rate is supposed to fluctuate with the volatility of the task structure and is therefore adaptive^62^. Finally, the adaptive prediction error (our main readout of the model) reflects how much the prediction about cue–outcome contingencies is updated due to the actual outcome of each trial, that is, the extent to which participants learned from their errors. In other words, the adaptive prediction error reflects the change in the subjective appreciation of the cues that will effectively translate to a shift of subsequent choices^24,28^.

#### General task performance does not depend on insulin sensitivity or liraglutide

Across the two groups (IS^+^ and IS^–^), participant task performance did not significantly differ concerning the proportion of correct trials (accuracy) or invalid trials (Table 1). Furthermore, both groups demonstrated the expected patterns in their general learning performance. Foremost, more predictable outcomes were consistently associated with smaller prediction error magnitudes (Supplementary Table 1; all *P* < 0.001), demonstrating that the participants could successfully learn the presented sensory contingencies. Additionally, the learning rate depended on the variance of the predictability (that is, volatility; see Supplementary Table 1e, effect of variance of predictability on learning rate, *F*_1,622_ = 92.92, *P* < 0.0001), indicating that the applied associative learning task successfully induced an adaptive learning process expressed by the adaptive learning rate of our model. Participants also showed more extensive behavioural updates after trials with correct predictions of the visual outcome than those with incorrect predictions (see main effects of correctness in Supplementary Table 1d,f; all *P* < 0.01). However, neither this effect nor the effect of predictability interacted with group or intervention in any analyses (all *P* > 0.140; Supplementary Table 1d,e), indicating that they can be interpreted independently of the group and drug interventions and confirming that the general learning pattern was consistent across conditions. Therefore, predictability and correctness were considered as factors of no interest in the following analyses.

**Table 1:** Characteristics of participants with normal (IS^+^) and impaired (IS^–^) insulin sensitivity under liraglutide and placebo conditions, respectively

Parameter	IS^+^ placebo	IS^+^ liraglutide	IS^–^ placebo	IS^–^ liraglutide	Main effect of group	Main effect of intervention	Interaction group × intervention
**Anthropometric data**							
Self-reported sex (female:male)	8:9	9:9	11:6	10:6	*X*^2^_3,68_ = 1.61		
Age (years)	26.29 (1.12)	26.89 (1.24)	26.58 (1.09)	25.31 (0.89)	*F*_1,41_ = 0.06 *P* = 0.798	*F*_1,23_ = 37.95*** *P* < 0.001	*F*_1,23_ = 0.00 *P* = 0.997
BMI (kg m^–2^)	24.36 (1.28)	24.16 (1.21)	33.72 (1.66)	32.91 (1.64)	*ψ* = 10.76** *P* = 0.006	*ψ* = 0.06 *P* = 0.673	*ψ* = 0.075 *P* = 0.8775
**Blood parameters and hunger rating**							
Insulin (mU liter^–1^)	6.19 (0.50)	8.58 (0.97)	14.00 (1.41)	20.64 (3.32)	*ψ* = 6.15* *P* = 0.031	*ψ* = 2.7* *P* = 0.027	*ψ* = −0.4 *P* = 0.278
Glucose (mmol l^–1^)	5.52 (0.1)	4.44 (0.07)	4.79 (0.08)	4.34 (0.07)	*F*_1,41_ = 3.12 *P* = 0.085	*F*_1,23_ = 32.8*** *P* < 0.001	*F*_1,23_ = 3.79 *P* = 0.064
HOMA-IR	1.23 (0.09)		3.01 (0.33)		*t*_18.6_ = 5.14*** *P* < 0.001		
Hunger	0.46 (0.07)	0.46 (0.05)	0.50 (0.07)	0.42 (0.06)	*F*_1,41_ = 0.03 *P* = 0.870	*F*_1,23_ = 0.87 *P* = 0.360	*F*_1,23_ = 0.42 *P* = 0.524
Nausea, 0800 h	0.16 (0.05)	0.19 (0.05)	0.17 (0.05)	0.17 (0.06)	*F*_1,41_ = 0.06 *P* = 0.811	*F*_1,21_ = 0.49 *P* = 0.491	*F*_1,21_ = 0.24 *P* = 0.629
Nausea, 0900 h	0.16 (0.06)	0.14 (0.04)	0.11 (0.03)	0.09 (0.03)	*F*_1,40_ = 2.09 *P* = 0.155	*F*_1,20_ = 0.41 *P* = 0.529	*F*_1,20_ = 0.002 *P* = 0.959
Nausea, 1000 h	0.16 (0.05)	0.08 (0.03)	0.09 (0.04)	0.05 (0.02)	*F*_1,40_ = 1.92 *P* = 0.173	*F*_1,23_ = 1.86 *P* = 0.185	*F*_1,23_ = 0.52 *P* = 0.479
**Task performance**							
Accuracy	0.77 (0.01)	0.76 (0.01)	0.77 (0.01)	0.78 (0.01)	*F*_1,41_ = 0.23 *P* = 0.632	*F*_1,23_ = 0.06 *P* = 0.816	*F*_1,23_ = 0.42 *P* = 0.525
Invalid trials	6.11 (2.48)	8.28 (2.64)	6.00 (2.23)	2.50 (0.78)	*ψ* = −1.5 *P* = 0.233	*ψ* = 0 *P* = 1	*ψ* = 1 *P* = 0.472
*ζ*	1.00 (0.12)	1.07 (0.14)	1.24 (0.18)	1.29 (0.21)	*ψ* = 0.43 *P* = 0.4172	*ψ* = 0.12 *P* = 0.289	*ψ* = 0.02 *P* = 0.845
*κ*	2.98 (0.33)	2.98 (0.27)	2.51 (0.32)	3.01 (0.28)	*ψ* = 0.74 *P* = 0.152	*ψ* = 0.41 *P* = 0.304	*ψ* = 0.93 *P* = 0.183
*ϑ*	0.0023 (0.00049)	0.0029 (0.00048)	0.0024 (0.00040)	0.0020 (0.00047)	*ψ* = 0.64 *P* = 0.882	*ψ* = 0.07 *P* = 0.495	*ψ* = 1.19 *P* = 0.362
**fMRI motion parameters**							
FD_max_	1.01 (0.26)	0.98 (0.17)	0.96 (0.11)	0.94 (0.14)	*F*_1,41_ = 2.55 *P* = 0.117	*F*_1,23_ = 6.39* *P* = 0.019	*F*_1,23_ = 0.04 *P* = 0.837

Descriptive statistics are presented on the left side and show means with s.e.m. in brackets. Inferential statistics are presented on the right side and vary depending on scales and normal distributions of the data. For normally distributed residuals, we report the *F*-test statistic. For non-normally distributed residuals, we report the test statistic *ψ* based on a robust mixed effect model. There was no significant interaction of group × intervention for any of the parameters (*P* > 0.2) except a trend for glucose (*P* = 0.06). Accuracy indicates the proportion of correct trials. Hunger and nausea (the latter determined at three timepoints: 0800, 0900 and 1000 h) were assessed using a visual analogue scale and were normalized so that the values ranged from 0 to 1. Invalid trials are reported as mean number of invalid trials out of 320. *ζ* represents encoding decision noise, *κ* determines how much the estimated environmental volatility affects the learning rate at the second level (coupling between the third and second levels), and *ϑ* determines the speed of learning about the log volatility of the environment (third level). FD_max_ is the maximal framewise displacement in millimetres as an index of head motion during fMRI.

*P < 0.05; **P < 0.01; ***P < 0.001.

#### Liraglutide normalizes dysregulated behavioural updating in insulin-resistant humans

**Fig. 2:**
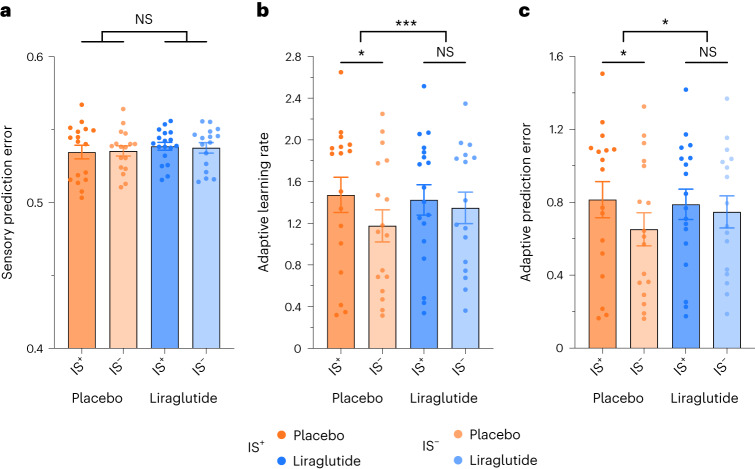
Differential effect of liraglutide on trial-wise measures of learning. **a**–**c**, While leaving sensory prediction errors (**a**) unchanged, liraglutide normalizes the adaptive learning rate (**b**) and adaptive prediction error (**c**) in individuals with impaired insulin sensitivity to the level of insulin-sensitive individuals. Data are presented as mean ± s.e.m. Data were analysed by mixed effect models with post hoc tests using Tukey’s procedure to test for the effects of intervention (placebo versus liraglutide) and group (IS^+^ versus IS^–^) on the respective learning parameter (IS^+^: placebo *n* = 17, liraglutide *n* = 18; IS^–^: placebo *n* = 17, liraglutide *n* = 16); *P < 0.05; ***P < 0.001; NS, not significant. Original *P* values are provided in Supplementary Table 1a–f. Source data

We then assessed whether the formation of the prediction errors differed between participants with impaired insulin sensitivity (IS^–^) and those with normal insulin sensitivity (IS^+^) and between the two interventions (liraglutide and placebo). Analysis of sensory prediction errors did not reveal any significant difference between the insulin-sensitive group and the group with impaired insulin sensitivity under placebo conditions (Supplementary Table 1a; *t*_332.82_ = −0.01, *P* = 0.993). Assessing the effects of intervention (liraglutide versus placebo), we neither found main effects nor an interaction with group (IS^+^ versus IS^–^; all *P* > 0.2; Fig. 2a and Supplementary Table 1d).

By contrast, the learning rate was significantly lower in the IS^–^ group than in the IS^+^ group under the placebo condition (Fig. 2b and Supplementary Table 1b; *t*_98.84_ = 2.24, *P* = 0.027), indicating a decreased adaptation of learning to predictability variations in individuals with impaired insulin sensitivity. Interestingly, the liraglutide intervention differentially affected the adaptive learning rate in the IS^+^ and IS^–^ groups as indicated by the interaction between group and intervention (*F*_1,626.2_ = 14.49, *P* < 0.001; Supplementary Table 1e). Post hoc tests indicated that while liraglutide enhanced the learning rate in the group with impaired insulin sensitivity (*t*_641.2_ = −6.48, *β* = −0.32, *P* < 0.001), the learning rate was reduced after GLP-1 agonistic intervention in the group with normal insulin sensitivity (*t*_640.0_ = 3.22, *β* = 0.15, *P* = 0.008). Notably, the effect of the intervention was twice as large in the group with impaired insulin sensitivity than in the group with normal insulin sensitivity. These opposing effects of liraglutide resulted in a convergence of the two groups’ adaptive learning rates so that they did not show any significant difference with liraglutide treatment (*t*_44.0_ = 0.53, *P* = 0.953).

As the adaptive learning rate scales the sensory prediction error to yield the adaptive prediction error, the amplitude of behavioural updating was also lower in the IS^–^ group than in the IS^+^ group with placebo treatment (Fig. 2c and Supplementary Table 1c; *t*_321.03_ = 2.50, *P* = 0.013) and was also differentially modulated by liraglutide in the IS^+^ and IS^–^ groups as indicated by the interaction between group and intervention (Supplementary Table 1f; *F*_1,620_ = 5.77, *P* = 0.017). Post hoc tests revealed that liraglutide significantly enhanced the amplitude of the adaptive prediction error in the group with impaired insulin sensitivity (Supplementary Table 1f; post hoc: *t*_650.8_ = −4.03, *P* < 0.001) but did not affect behavioural updating in the insulin-sensitive group (*t*_649.1_ = 2.10, *P* = 0.155). As a consequence, the adaptive prediction error did not differ between the two groups (IS^+^ and IS^–^) under the liraglutide condition (*t*_48.8_ = −0.58, *P* = 0.939), indicating that liraglutide was able to restore adaptive prediction error encoding of people with low insulin sensitivity to the level of those with high insulin sensitivity. Taken together, these results reveal that insulin resistance is associated with an impairment of associative learning, which the application of liraglutide can mitigate.

### fMRI data analysis

To assess the neural responses underlying the differential effects of liraglutide intervention on learning depending on peripheral insulin sensitivity, we analysed the fMRI data to identify brain regions encoding adaptive prediction errors and studied whether liraglutide intervention (relative to placebo) enhanced this neural encoding to a more substantial extent in the group with impaired insulin sensitivity than in the insulin-sensitive group.

#### Adaptive prediction errors are encoded in the NAc and ventromedial prefrontal cortex

First, we tested for brain regions that encode adaptive prediction error. Reproducing the results of prior work^59^, adaptive prediction error evoked prominent activations in the NAc, putamen, mid-insula and ventromedial prefrontal cortex (vmPFC; Supplementary Table 2), confirming that adaptive prediction error encoding during sensory associative learning primarily recruits corticostriatal, putatively dopaminergic pathways^28,63^.

#### Liraglutide upregulates adaptive prediction error encoding in the subcallosal area and the NAc

**Fig. 3:**
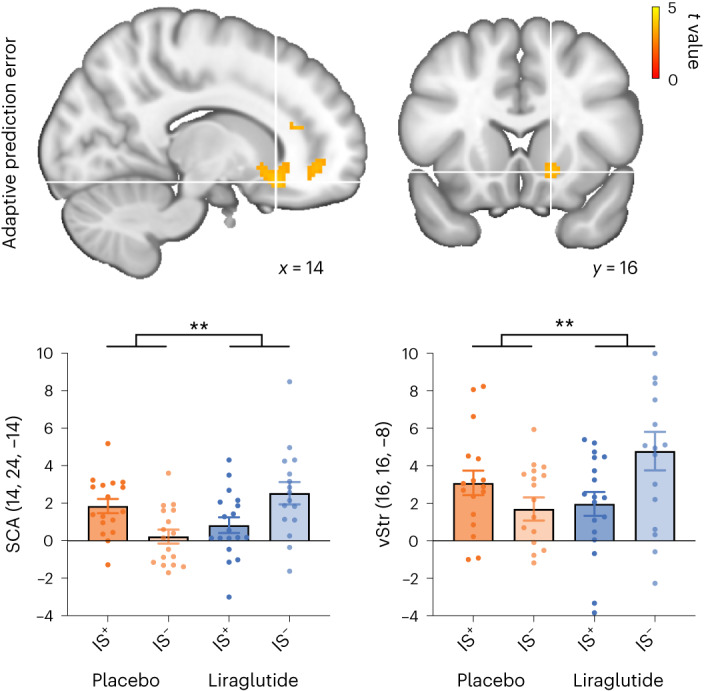
Liraglutide enhances learning-related brain activity in individuals with impaired insulin sensitivity. The interaction group × intervention identified brain regions in which the encoding of adaptive prediction errors was more strongly enhanced by liraglutide than placebo in the insulin-resistant group (IS^–^) than in the insulin-sensitive group (IS^+^). Liraglutide enhanced adaptive prediction error encoding in the IS^–^ group in the SCA and the ventral striatum (vStr). Activation maps were overlayed on the standard brain atlas provided by the Montreal Neurological Institute (MNI) (the statistical threshold was *P* < 0.05, with data family-wise error corrected at the cluster level and with an underlying voxel-level threshold of *P* < 0.001). Statistical analyses were conducted using Statistical Parametric Mapping version 12 in the framework of a general linear model (GLM) with flexible factorial designs. Bars and error bars correspond to the mean and s.e.m. of the contrast estimates at the peak of the cluster inferred at the group level, reflecting the relationship between trial-wise BOLD responses to visual outcomes and adaptive prediction errors. The corresponding activation peaks are marked with white crosses, with respective anatomical labels and MNI coordinates (*x*, *y* and *z*) indicated in each plot. Data points correspond to the individual contrast estimates at the same voxel (IS^+^: placebo *n* = 17, liraglutide *n* = 18; IS^–^: placebo *n* = 17, liraglutide *n* = 16); **P < 0.01. Source data

As our behavioural analysis revealed that liraglutide intervention significantly enhanced the amplitude of adaptive prediction error encoding in the group with impaired insulin sensitivity, we specifically tested for brain regions in which liraglutide (relative to placebo) enhanced the neural encoding of adaptive prediction error to a greater extent in the group with impaired insulin sensitivity than in the group with normal insulin sensitivity. In the vmPFC and the ventral striatum extending to the NAc, the encoding of adaptive prediction errors was indeed enhanced by liraglutide relative to placebo only in individuals with low insulin sensitivity but not in those with high insulin sensitivity (Table 2 and Fig. 3). This finding confirms that liraglutide intervention combined with impaired insulin sensitivity enhances adaptive prediction error encoding, thus agreeing with the behavioural results. Notably, the inverse interaction effect (greater liraglutide-driven adaptive prediction error encoding in the IS^+^ group than in the IS^–^ group) did not reveal significant activations.

**Table 2:** Differential effects of liraglutide on neural tracking of the adaptive prediction error

	Cluster level		Peak level			
	*P*_FWE-corr_	Size	*t*	*x*	*y*	*z*
**Interaction**						
dACC	0.004	704	4.69	18	38	16
lOFC			4.21	26	48	−12
SCA			3.97	14	24	−14
vmPFC			3.95	16	42	−10
vStr/NAc			3.71	16	16	−8
**IS**^+^**: liraglutide** **>** **placebo**						
No significant clusters						
**IS**^**–**^**: liraglutide** **>** **placebo**						
vmPFC	0.001	342	4.67	16	42	−14
lOFC			3.79	30	46	−12
dACC	0.033	12	4.05	18	38	16
SCA	0.013	71	3.97	14	28	−16
vStr/NAc			3.72	16	16	−8

Statistics for *t*-contrasts identifying brain regions in which liraglutide (relative to placebo) enhanced the encoding of learning to a greater extent in the group with impaired insulin sensitivity (IS^–^) than in the group with normal insulin sensitivity (IS^+^). In addition to this interaction effect, we tested for the liraglutide effect within the IS^+^ and IS^–^ groups, respectively, masked with the corresponding interaction result. The statistical threshold was *P* < 0.05, and data were family-wise error corrected at the cluster level (*P*_FWE_-corr), with an underlying voxel-level threshold of *P* < 0.001. Corresponding brain activation maps are shown in Fig. 3.

dACC, dorsal anterior cingulate cortex; lOFC, lateral orbitofrontal cortex.

Together, the behavioural and fMRI results indicate that liraglutide normalizes learning in individuals with impaired insulin sensitivity by enhancing adaptive prediction error encoding in the ventral striatum and its mesocortical projection sites. We thereby demonstrate a modulatory role of bodily metabolic feedback signals on prediction error encoding, rendering the underlying neural circuit function vulnerable to insulin sensitivity and affected by physiological signals from the periphery, such as GLP-1.

## Discussion

Arguing that the motivational force prompting behavioural adaptation must ultimately rely on learned sensory associations, we scrutinize a general role for metabolic sensing in associative learning. Here, our main hypothesis rests on the assumption that metabolic signals from the periphery affect DA neuron function in the mesoaccumbens pathway and hence alter learning. We further consider impaired insulin sensitivity of the DA midbrain as a possible cause for impaired learning of sensory associations and investigate whether augmenting the endogenous metabolic feedback signal with the GLP-1 receptor agonist liraglutide normalizes associative learning. By applying a generative model with sufficient hierarchical depth to handle events that unfold at different spatial and temporal scales and that have already been established to provide reliable estimates of individual learning performance^59,60,63^, we evaluated three computational learning parameters: (1) a sensory prediction error weighted by the (2) adaptive learning rate to yield the (3) adaptive prediction error as a measure of behavioural updating.

Our behavioural results revealed similar patterns of sensory prediction error formation under placebo and intervention conditions irrespective of insulin sensitivity. Participants with impaired insulin sensitivity exhibited a reduced amplitude of behavioural updating, which was normalized by the liraglutide intervention. This effect of the GLP-1 receptor agonist on behavioural updating is driven by the enhanced encoding of adaptive prediction errors in the ventral striatum and its cortical projection sites, including prefrontal (vmPFC) and orbitofrontal cortices related to the adaptive encoding of decision variables^64,65^, the anterior cingulate cortex regarding learning under uncertainty and adaptive hypothesis formation^66^ and the subcallosal area (SCA)^67^. The latter is ideally located for rendering learning processes susceptible to metabolic sensing of an interoceptive state as it receives afferents from various cortical structures (the PFC, orbitofrontal cortex and anterior cingulate cortex), basolateral amygdala, hippocampus, thalamus, (dorsal and lateral) hypothalamus, VTA and raphe^68^, that is, hallmark regions recently widely discussed to render underlying learning processes susceptible to metabolic sensing of interoceptive states^11,33,34^ and the postingestive effects of food^35,36^.

Notably, the learning task used did not involve explicit reinforcement (food or any other reward) but merely sensory cues. Thus, the documented regulation of prediction error learning by metabolic signals is not restricted to learning about homeostatically relevant cue–outcome associations. Interestingly, midbrain dopaminergic neurons are capable of facilitating associations between contiguously occurring events, regardless of the content of those events^69^. That is, any interaction with an error signal that is encoded by these neurons will affect learning and not just reward learning.

Our results support a growing body of literature indicating that metabolic signals profoundly influence neuronal processing (particularly those signalling energy restoration in an energy deprivation scenario, such as insulin and GLP-1 signalling a postprandial state in our participants after an overnight fasting period). Fundamental neural processes, such as coding precision of visual information in the neocortex^70^ or motivation to exert physical effort for food and food-independent rewards^48^, were recently shown to be regulated by the metabolic state.

We further show that impairment of metabolic signalling (such as impairment of insulin sensitivity in obesity) causes deficiencies in associative learning. In line with this finding, previous studies revealed impaired outcome learning in obesity^55,56^, suggesting that learning impairments in obesity likely relate to reduced metabolic signalling in mesoaccumbens pathways. Here, we show that these deficiencies are restored by liraglutide intervention without causing side effects. Besides obesity, this restorative potential of GLP-1 analogues has implications for other pathologies characterized by impaired dopaminergic function and associated with metabolic impairments, such as psychosis^71^, Parkinson’s disease^72^, depression^73^ or even cardiovascular events^74^.

Regarding the question of which pathway the peripherally administered GLP-1 receptor agonist liraglutide recruits to affect DA function in the mesoaccumbens pathway, we currently lack evidence that liraglutide enters the VTA or NAc. Ultimately, vagal afferents may be relevant for systemic GLP-1 sensing^75–77^. Accordingly, peripherally administered liraglutide has been detected in the solitary nucleus (NTS)^78^, the primary sensory vagal projection nucleus in the brainstem; hence, it most likely affects mesostriatal DA signalling indirectly by binding to GLP-1 receptor-expressing glutamatergic or GABAergic neurons^78^ or astrocytes^79,80^ within the NTS. These neurons regulate the GLP-1-expressing neurons of the NTS, which project to the VTA, suppressing activity of DA neurons in the mesoaccumbens pathway^46^, and might consequently affect learning. Alternatively (or additionally), access via leaks in the circumventricular organs (area postrema, median eminence) might secondarily influence the parabrachial nucleus and the central nucleus of the amygdala as well as the bed nucleus of the stria terminalis^81^ and thereby indirectly affect signalling of the DA mesostriatal system.

### Limitations

Although our behavioural and fMRI results are remarkably compatible with the above-presented animal data^43–46^ on neural processes affected by metabolic sensors and previous behavioural human data^56^, the proposed molecular mechanisms and central access routes of peripheral liraglutide remain speculative in humans. Furthermore, as liraglutide increases insulin secretion leading to a slight reduction in peripheral glucose levels (Table 1), the observed effect cannot currently be attributed to liraglutide without considering an overlapping effect of liraglutide and insulin. Moreover, our study design does not allow for testing of meaningful sex differences in metabolic regulation of prediction error encoding. Also, the dichotomization approach that we have chosen to stratify participants into insulin-sensitive and insulin-impaired groups (IS^+^ and IS^–^) might be disadvantageous compared to using HOMA-IR as a continuous variable. However, even if we would assume a linear, continuous increase in insulin sensitivity in the sample, the dichotomization will just decrease the statistical power; we have here chosen the safest approach to the problem. A further noteworthy caveat comprises the inability of the applied hierarchical model to capture more subtle effects of metabolic signalling on dopaminergic learning processes, such as the modulation of differential learning from positive and negative prediction errors. The detection of these differential effects would, however, require a new generation of learning models combining an adaptive learning rate and bias registration to consider asymmetrical learning.

Collectively, our behavioural and fMRI findings reveal that GLP-1 receptor activation normalizes associative learning in insulin-resistant humans by modulating the encoding of adaptive prediction errors within the mesoaccumbens pathway, indicating that DA-driven learning processes depend on metabolic signalling, and this may contribute to the weight-reducing effects of liraglutide in obesity.

## Methods

### Participants

Fifty-four healthy volunteers with a large variance in body weight (Table 1) were recruited for this study given a power analysis assuming an *α* (significance) value of 0.05, a power of 0.95 and a medium effect size relating to a Cohen’s *d* of 0.5. The power estimation (G*Power version 3.1) was performed assuming a mixed effect model with repeated measurements in two groups (normal versus impaired insulin sensitivity) and considering within- and between-group interactions (liraglutide versus placebo), yielding a total sample size of *N* = 54.

**Extended Data Fig. 1:**
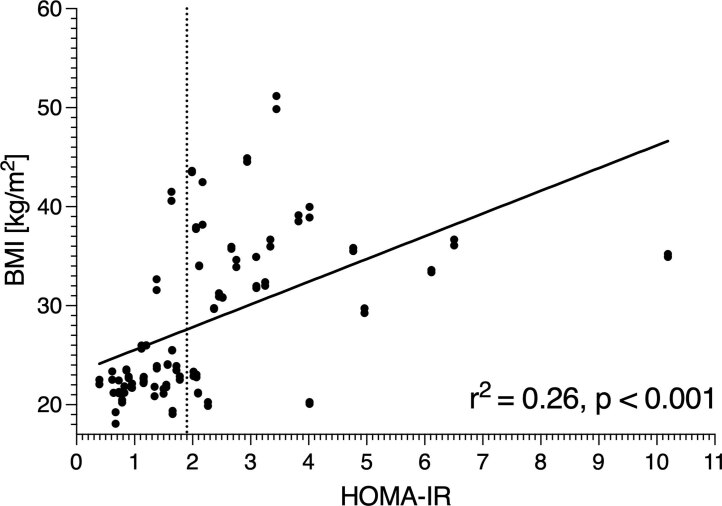
Correlation between BMI and HOMA-IR. Within the study participants, BMI and HOMA-IR were highly correlated; *r*^2^ = 0.26, *P* < 0.001 based on the Pearson’s correlation test. Source data

All participants were recruited from the preexisting database of volunteers maintained at the Max Planck Institute for Metabolism Research. Participants were medication-free non-smokers without any history of neurological, psychiatric, gastrointestinal or eating disorders and without any special diets. To assess each participant’s peripheral insulin sensitivity as a proxy for their central insulin sensitivity, we considered the HOMA-IR^58^. More precisely, we calculated the HOMA-IR of each participant as (fasting serum glucose (mmol l^–1^) × fasting serum insulin (mU liter^–1^))/7,290 on the placebo day, with lower values indicating a higher degree of insulin sensitivity; the HOMA-IR was calculated only for the placebo day, as GLP-1 analogues may increase insulin secretion and alter the HOMA-IR. To then stratify groups according to normal and impaired insulin sensitivity, participants were assigned to an insulin-sensitive group (IS^+^) if their HOMA-IR was ≤1.9 and to a less insulin-sensitive/insulin-resistant group (IS^–^) if their HOMA-IR was >1.9. Note that BMI highly correlates with HOMA-IR (*r* = 0.5, *t* = 4.670, d.f. = 66, *P* ≤ 0.001); therefore, it is not possible to separate variance due to adiposity versus altered insulin sensitivity (Extended Data Fig. 1), and we attended only to HOMA-IR as a group-defining factor in our data analysis.

All participants performed one session of an associative sensory learning task (for a detailed task description, see Iglesias et al.^59^) during fMRI on two separate days, with a GLP-1 analogue (see below) administered on the first day and placebo on the second day. Individual sessions were excluded from data analysis based on elimination criteria regarding task performance and excessive head motion to avoid artefacts in fMRI data. In total, 40 sessions (20 placebo sessions and 20 liraglutide sessions) were excluded due to the following reasons: 5 sessions had more than 20% invalid trials (missing response or a response later than 1.5 s), 17 sessions had less than 65% accuracy, 6 sessions had participants always pressing the same button, 1 session had technical problems, 1 session had a computational model that could not be fitted, and 10 sessions had excessive head motion (FD_max_ > 4 mm).

As a result, a total of 68 individual sessions (34 placebo sessions and 34 liraglutide sessions) from 43 different participants (23 in the insulin-sensitive group and 20 in the insulin-resistant group) were included into the analysis (see Table 1 for sample characteristics). In other words, we could include both sessions for 25 participants and one session for 18 participants and had to exclude both sessions for 11 participants.

The final sample (*N* = 43 with 68 included sessions) allowed for a power of 0.62 for the endpoint of our model analysis (adaptive prediction error) given a two-way interaction of insulin sensitivity (normal versus impaired) × intervention (liraglutide versus placebo) within the used mixed effect models, relating to effect sizes of Cohen’s *f* of 0.1 and 0.15, respectively, at a significance level *α* = 0.05.

All participants gave written informed consent to participate in the experiment, which was approved by the local ethics committee of the Medical Faculty of the University of Cologne (16-251). In addition, the study has been prospectively registered at https://clincalsite.org (ID 2688).

### Study procedure

The study was performed in a single-blinded, placebo-controlled, randomized crossover design. Each volunteer participated on two testing days lasting a maximum of 2 h each. Both testing days were separated by a minimum of 1 week to allow for a sufficient washout period^82^. The order of the intervention (liraglutide versus placebo) was counterbalanced (Fig. 1a).

The evening before each testing day, participants first received either an agonistic GLP-1 analogue (see below) or an equal volume of saline solution, followed by a standardized dinner with equal caloric amounts per individual (Supplementary Table 3). The next morning, participants arrived fasted at the institute at 0800 h, and their BMI was measured using the seca mBCA 515 (medical body composition analyser). As this study was part of a larger study, all participants underwent not only the fMRI task as detailed below but also an incentivized motivation task in a purely behavioural session that was related to a different research question and is reported by Hanssen et al.^48^. The order of the fMRI task and the behavioural task was counterbalanced. Before each task, hunger levels were assessed via visual analogue scales, and a blood draw was taken to measure insulin and glucose levels (Fig. 1b). All measurements were performed between 0800 and 1000 h.

### GLP-1 analogue

A subcutaneous injection of 0.6 mg of liraglutide (Novo Nordisk) was used as an agonistic GLP-1 analogue. As the maximum plasma concentration of liraglutide is reached approximately 11–13 h after injection^82^, liraglutide was administered the evening before the testing day between 1900 and 2000 h to assure sufficient levels at the start of the testing day. Under the placebo condition, an equal volume of saline solution was injected subcutaneously.

### Hunger ratings

To control for differences in hunger states between testing days, we instructed the participants to rate hunger before the task on each testing day using a visual analogue scale, as described previously^83^. In brief, on a 100-mm visual analogue scale (0 indicates ‘gar nicht hungrig’ (not hungry at all), and 100 mm indicates ‘sehr hungrig’ (very hungry)), participants were asked to mark the point that most accurately represented their perception of their current hunger state^48^.

### Insulin and glucose levels

As GLP-1 was reported to increase insulin secretion^84^, we monitored insulin to control for insulin effects at the onset of the task. To this end, we took blood samples directly before starting the task and measured the levels of insulin and glucose.

### Experimental design: associative learning task

Participants performed the same associative learning task as described previously (fMRI study 2 by Iglesias and colleagues^59,63^). In brief, the task is, by design, cross-modal (audiovisual), meaning that participants have to learn the predictive strength of auditory cues to predict which of two possible visual target categories would subsequently follow.

At the beginning of each trial, participants heard either a high tone (576 Hz) or a low tone (352 Hz). After this cue, they had to predict whether the upcoming picture would be a face or a house by a button press (index or middle finger), providing us with an explicit behavioural readout of their prediction. The appearance of the picture gave participants explicit feedback about the correctness of their predictions and allowed them to update their beliefs trial by trial. There were no trial-wise monetary rewards; participants only received a fixed monetary compensation for participating in the study, which was independent of their task performance.

The cues were presented for 300 ms, the response interval was 1,200 ms, the duration of the visual outcome presentation was 300 ms, and the intertrial interval varied randomly between 1.5 and 2.5 s. Importantly, the cue–outcome association strength varied over the blocks of the task (volatility), including cues that were highly predictive (probability of 0.9 for a face and 0.1 for a house or vice versa), moderately predictive (probability of 0.7 for a face and 0.3 for a house or vice versa) and non-predictive (probability of 0.5). Participants were not informed about the sequence of probabilities^59^.

On each testing day, participants completed 320 trials, which were divided into ten blocks of different predictive strengths. Both block length (24 or 40 trials) and sequence of probabilities varied randomly across blocks. Before the task, participants underwent a psychophysical matching to adapt the volumes of the two auditory cues (high and low tone) so that they perceived both tones as equally loud^85^. The task was presented using the Cogent2000 graphics toolbox for Matlab. (Note: This toolbox has recently been replaced by the Psychtoolbox: http://psychtoolbox.org.)

### Computational modelling of behavioural data

For analysis of the behavioural data from the learning task, we modelled the trial-by-trial changes in participants’ choices with the HGF^61,86^, yielding participant-specific parameter estimates and learning trajectories estimates. Unlike more classical learning models, the HGF does not assume a fixed learning rate but allows for an online adaptation of the learning rate as a function of the volatility of the cue–outcome associations^60^. To that end, the HGF contains coupled hierarchical levels continuously adapting the individual learning progress. We refer the reader to Iglesias et al.^59^ as well as to Mathys et al.^61^ for the mathematical details of the model; here, we will only briefly introduce its general concepts. Furthermore, for the sake of simplicity and to keep the focus on the associative learning mechanism per se, we use in this manuscript a different notation than in the original papers but put in quotes, for reference, the original terminology.

The first level of the HGF simply represents the occurrence of the auditory and visual stimuli (that is, perception of the ‘stimulus category’). The second level captures the learning of the conditional probabilities of the visual stimulus given the auditory cue. This learning involves the formation of a sensory prediction error (the ‘low-level choice prediction error’ in the HGF), which is then weighted with an adaptive learning rate (the ‘precision weight’ at the second level in the HGF) to construct an adaptive prediction error (the ‘low-level precision-weighted choice prediction error’ in the HGF) that, in turn, drives behavioural updating. The third level of the HGF tracks the ‘log volatility of the environment’; that is, it reflects the rate of change of the contingencies and therefore the need to decrease or increase the rate of learning in the lower level (for a formal derivation, see Supplemental Experimental Procedures, Section A, by Iglesias et al.^59^).

In this study, our main hypothesis rests on the assumption that metabolic signals from the periphery control DA neuron function in the mesoaccumbens pathway and therefore will modulate the learning of cue–outcome associations. Thus, based on mounting evidence for adaptive prediction error encoding in the ventral striatum and midbrain^26,28^ and recent findings related to the HGF^59^ suggesting that ‘low-level prediction errors’ activate the ventral striatum (signed prediction errors) and midbrain (absolute prediction errors), high-level uncertainty tracking in the HGF instead relates to other neuromodulatory systems (cholinergic in particular). Accordingly, we ignored the third level of the HGF and restricted our behavioural analyses to the lower-level computational quantities recovered by the model.

For the analysis, we used the implementation of the HGF as introduced by Iglesias et al.^59^, specifically model hgf_3l_ (ref. ^63^), and provided in the TAPAS toolbox (version 1.0; https://www.translationalneuromodeling.org/tapas). By fitting the choice data of each participant for each session, we estimated participant-specific trajectories of three different computational quantities.

1. The sensory prediction error about the visual outcome in a given trial, which corresponds to the ‘low-level choice prediction error’ in the HGF (see Supplemental Experimental Procedures, Section B, by Iglesias et al.^59^). Ultimately, the prediction error relates to the difference between the actual correctness of the participant’s choice and their subjective expectation (in terms of a priori probability) of this choice being correct.
2. The adaptive learning rate, which corresponds to the ‘precision weight’ at the second level in the HGF^86^.
3. The adaptive prediction error, that is, the product of the sensory prediction error (i) and the adaptive learning rate (ii). The adaptive prediction error relates to the ‘precision-weighted choice prediction error’ about the visual outcome in the HGF and was used here as parametric modulator for the subsequent fMRI analysis.

Note that in the original HGF formulation, the second-level update equation capturing associative learning relies on (‘low-level’) prediction errors, which are encoded in the perceptual reference frame and are unsigned. However, prediction errors can be seamlessly re-encoded in the action space, yielding a signed (‘low-level choice’) prediction error, positive when the participant made a correct choice and negative when the participant made an incorrect choice^59^. As our question relates to the brain regions implementing behavioural updates, we adopted the latter approach in our analyses; the sensory prediction error and the adaptive prediction error are therefore signed and relate to the ‘low-level choice prediction error’^59^. This allowed us in our fMRI analysis to keep in line with previous literature on prediction error encoding in the mesoaccumbens circuitry. For the behavioural analysis, as we were interested in whether or not participants learn, we also considered the absolute prediction error to measure the amplitude of updating^57^.

### Statistical inference

All behavioural, blood and anthropomorphic data were analysed using RStudio (version 1.4.1717) and R (version 4.0.0). First, we performed normality testing using ‘Q-Q plots’ in R. If residuals were normally distributed, analysis of variance (ANOVA) tests based on mixed effect models (test statistic *F*) were implemented using the ‘lme4’ R package (version 1.1-26) together with the ‘lmerTest’ R package (version 3.1-3) for a denominator d.f. approximation and significance tests. In these mixed effect models, we used group (normal insulin sensitivity (IS^+^) versus impaired insulin sensitivity (IS^–^)) and intervention (liraglutide versus placebo) as fixed effects and participant ID as a random intercept (Table 1); we also considered possible interactions between group and intervention. If residuals were not normally distributed, ANOVAs with bootstrapping based on robust mixed effect models were implemented using the Wilcox WRS functions (version 1.1-0) implemented in R (test statistic *ψ*). Power analyses were performed in G*Power (version 3.1).

Considering the above-discussed computational states of learning behaviour (that is, sensory prediction error, adaptive learning rate and adaptive prediction error), we first tested whether impaired insulin sensitivity in participants impacts learning behaviour. Here, we applied independent *t*-tests separately for each of the computational quantities to test for differences between the IS^+^ and IS^–^ groups under placebo conditions (Supplementary Table 1a–c).

To scrutinize for the effect of liraglutide intervention, we first assessed the effects of group and intervention on sensory prediction error as a basic component of learning. To account for other task-dependent features that changed from trial to trial, we additionally included correctness (incorrect or correct participant response) and predictability (the true probability for the visual outcome dependent on the current cue–outcome association strength varying between 0.9, 0.7, 0.5, 0.3 and 0.1; see task description above). To simplify the analysis, we followed a summary statistics approach and computed a mean sensory prediction error separately for each participant and separately for all possible factor combinations (five predictability levels × two correctness levels). As introduced above, we used absolute sensory prediction errors in our behavioural model. This resulted in the following model:

$$\begin{array}{l}{{\mathrm{M}}}_{1}:{\rm{sensoryPredictionError}}\, \sim\\ \,1+{\rm{group}}\times {\rm{intervention}}\times {\rm{correctness}}\times {\rm{predictability}}+\left(1\,|{\rm{ID}}\right).\end{array}$$

We tested whether liraglutide (relative to placebo) affected the choice prediction error differently in the IS^–^ group compared to in the group of participants with normal insulin sensitivity (Supplementary Table 1d).

Thereafter, we tested the effect of intervention and group on the adaptive learning rate, which reflects the relative impact of sensory prediction error on behavioural updating. The adaptive learning rate is always positive and depends on the variance of the cue predictability (three instead of five levels) rather than on correctness and the predictability itself; thus, we used the following model (Supplementary Table 1e):

$$\begin{array}{l}{{\mathrm{M}}}_{2}:{\rm{adaptiveLearningRate}}\, \sim\\ \,1 + {\rm{group}}\times {\rm{intervention}}\times {\rm{variance}}\; {\rm{of}}\; {\rm{predictability}}+\left(1\,|{\rm{ID}}\right).\end{array}$$

Finally, we scrutinized the effect of group and intervention on the size of the adaptive prediction error, the result from the modulation of the sensory prediction error by the adaptive learning rate and the ultimate readout reflecting the size of the actual behavioural update. Note that here we also ignored the sign of the prediction error (see above); hence, in congruence with M_1_ and M_2_, the following model was applied (Supplementary Table 1f):

$$\begin{array}{l}{{\mathrm{M}}}_{3}:{\rm{adaptivePredictionError}}\, \sim\\ \,1+{\rm{group}}\times {\rm{intervention}}\times {\rm{correctness}}\times {\rm{predictability}}+\left(1\,|{\rm{ID}}\right).\end{array}$$

All post hoc analyses and comparisons were calculated using the Tukey’s procedure ‘lsmeans’ R package (version 2.30-0) for the group × intervention contrasts. Effect size measures (Cohen’s *d* or *f*) were calculated by using the R package ‘effectsize’ (version 0.7.0.5).

### fMRI acquisition parameters

All imaging was performed on a 3T MRI system with a 64-channel head coil (Siemens Magnetom Prisma Fit). The MRI data were acquired using a Magnetom Prisma^fit^ 3T whole-body scanner and a 64-channel head coil (Siemens AG, Medical Solutions). During the task, fMRI data were acquired in one session with a T2-weighted echo-planar imaging sequence (31 axial slices with a slice thickness of 2 mm, in-plane resolution of 2 mm × 2 mm, no distance factor, ascending interleaved in-plane acquisition, a repetition time (TR) of 2,000 ms, an echo time (TE) of 30 ms, a flip angle of 90° and a field of view of 224 × 224 × 60 mm^3^). This protocol did not cover the whole brain but focused on brain regions of interest, including the midbrain, striatum and vmPFC (16th slice on the anterior commissure–posterior commissure line). Functional data acquisition lasted 22.6 min and included 678 volumes. Two additional images (each including three volumes) were collected with the same fMRI protocol but with reversed phase-encoding directions, resulting in a pair of images with distortions going in opposite directions. High-resolution T1-weighted images were obtained from the institute’s subject database (Modified Driven Equilibrium Fourier Transform sequence: TR = 1,930 ms, TE = 5.80 ms, field of view = 256 × 256 × 160 mm^3^, voxel size = 1 × 1 × 1.25 mm^3^ and 128 sagittal slices; Magnetization-Prepared Rapid Gradient-Echo sequence: TR = 2.300 ms, TE = 2.32 ms, field of view = 256 × 256 × 192 mm^3^, voxel size = 0.9 × 0.9 × 0.9 mm^3^ and 213 sagittal slices).

### fMRI statistical analysis

The individual data sets were preprocessed before running statistical analyses using tools from the FMRIB Software Library (FSL version 5.08, https://www.fmrib.ox.ac.uk/fsl) and in accordance with Smith et al.^87^. Non-brain tissues (for example, scalp and cerebrospinal fluid) were removed using an automated brain extraction tool^88^. Time series were realigned to correct for small head movements using FSL’s MCFLIRT^89^. Susceptibility-induced distortions were estimated based on the two images with reversed phase-encoding directions using the TOPUP tool as implemented in FSL^90,91^ and used for distortion correction of the functional images. Data were spatially smoothed using an 8-mm full-width at half-maximum Gaussian kernel. Structured artefacts were then removed using an independent component analysis followed by FSL’s ICA-based X-noiseifier^92,93^. Functional data were then co-registered to the participant’s T1-weighted image and normalized to the MNI standard space.

Statistical analyses were conducted using Statistical Parametric Mapping version 12 (r6225, Wellcome Trust Centre for Neuroimaging) implemented in MATLAB R2019b (MathWorks) in the framework of a GLM. At the single-participant level, conditions were modelled using a boxcar reference vector convolved with the canonical hemodynamic response function and its time derivative^94^. For each session (liraglutide and placebo) of each participant, we created a first-level GLM to identify brain regions in which fluctuations in outcome-related activity correlated with trial-wise variations of adaptive prediction error encoding. In this GLM, the BOLD response to outcomes was parametrically modulated by the adaptive prediction error (as described above), separately for faces and houses^57^. In brief, although we were not interested in the differential brain responses related to learning from faces and houses, we modelled these two types of outcomes separately because the basic (learning-independent) processing of these two visual stimuli is well known to recruit distinct brain regions. Taken together, the GLM included the following regressors: cue (duration = 0.3 s), prediction (duration = 1.2 s), faces (duration = 0.3 s), parametric modulation of faces, houses, parametric modulation of houses, optionally trials with missing responses (spanning the entire trial, from cue to outcome) and nuisance regressors (six motion parameters relating to the current and preceding volumes, respectively, plus each of these matrices squared; see Friston et al.^95^). Low-frequency signal drifts were high-pass filtered using a cutoff of 128 s. To identify brain regions that encoded the adaptive prediction error regardless of the type of visual outcome, we computed contrast images that averaged the effects of parametric modulation by the adaptive prediction error across faces and houses. We then entered these contrasts into the group-level analysis.

At the second (group) level, we specified a GLM to investigate the effects of insulin sensitivity and intervention on adaptive prediction error neural encoding. In a flexible factorial design, the factors participant, group (normal insulin sensitivity (IS^+^) and impaired insulin sensitivity (IS^–^)) and intervention (placebo and liraglutide) were specified, with all variances set to unequal and dependency set to 1 for intervention and 0 otherwise. Because every participant performed the learning task differently, the time courses of learning trajectories were heterogeneous. As our analyses focused on the correlation between the fMRI BOLD response and precisely these learning trajectories (which can easily be influenced by outliers^96^), we used the correction for the resulting departures from sphericity by assuming unequal variance for the factor participant, making the inclusion of random participant blocks unnecessary^97–99^. The GLM included four regressors: IS^+^ placebo, IS^+^ liraglutide, IS^–^ placebo and IS^–^ liraglutide. We first used the conjunction of these four regressors to identify brain regions involved in adaptive prediction error encoding. We then aimed to identify brain regions in which liraglutide (relative to placebo) enhanced the neural encoding of adaptive prediction errors to a greater extent in the IS^–^ group than in the IS^+^ group (interaction group × intervention, contrast weights [1 −1 −1 1]). To test whether the interaction results were driven by the liraglutide (versus placebo) effect in just one of the two groups, we also computed pairwise comparisons within each group (contrast weights [−1 1 0 0] and [0 0 −1 1]); these were masked and small volume corrected with the respective interaction result. Group-level results were thresholded at *P* < 0.05 and family-wise error corrected at the cluster level, with a cluster-defining threshold of *P* < 0.001.

### Reporting summary

Further information on research design is available in the Nature Portfolio Reporting Summary linked to this article.

### Supplementary information

Supplementary Information Study protocol as approved by the Institutional Review Board (ethics committee of the Medical Faculty of the University of Cologne, number 16-251).

Reporting Summary

Supplementary Table 1 Test for the effects of intervention (placebo and liraglutide) and group (IS^+^ and IS^–^) on learning parameters, including post hoc tests.

Supplementary Table 2 Neural tracking of the adaptive prediction error.

Supplementary Table 3 Standardized dinner the evening before the testing day.

### Source data

Source Data Fig. 2 Data points used to generate Fig. 2.

Source Data Fig. 3 Data points used to generate Fig. 3.

Source Data Extended Data Fig. 1 Data points used to generate Extended Data Fig. 1.

## Data Availability

The human data reported in this study cannot be deposited in a public repository per General Data Protection Regulation and Institutional Review Board data protection policies. To request access, please contact the lead contact. Data provision may include processed and unprocessed data and will require a data-sharing agreement. Data sharing necessitates that the purpose of data reanalysis is in line with the study aims as approved by the ethics review boards and participant consent. Furthermore, consent to data privacy needs to be assured by signing the agreement form accordingly. Requests will be answered within 4 weeks. Source data are provided with this paper.
