# Supplementary material for: Liraglutide restores impaired associative learning in individuals with obesity
Source: Nat Metab. 2023 Aug 17;5(8):1352–63. doi: 10.1038/s42255-023-00859-y (PMC10447249; doi:10.1038/s42255-023-00859-y)
Supplement: Supplementary file 1 — Study protocol as approved by the Institutional Review Board (ethics committee of the Medical Faculty of the University of Cologne, number 16-251). [file 42255_2023_859_MOESM1_ESM.pdf]

nature metabolism

Article

https://doi.org/10.1038/s42255-023-00859-y

Liraglutide restores impaired associative learning in individuals with obesity

In the format provided by the authors and unedited

Antrag an die Ethik-Kommission der Medizinischen Fakultät der Universität Köln

A. FORMALES

**1 Title**	Prediction error learning and motivation in obesity: The physiological effect of Glp-1 receptor activation
**2 Applicants**	Prof. Dr. Jens Brüning[1,2], Dr. Ruth Hanßen[1,2], Kerstin Albus[3], Dr. Lionel Rigoux[1], Prof. Dr. Oliver Cornely[3], Dr. Michael Faust[2], Dr. Marc Tittgemeyer[1]<br><br>[1]MPI for Metabolism Research, Cologne<br>[2]Centre for Endocrinology, Diabetes and Preventive Medicine, University Hospital of Cologne<br>[3]CECAD, Translational Platform, University of Cologne
**3 Testing centre**	Centre for Endocrinology, Diabetes and Preventive Medicine, University Hospital of Cologne, Kerpener Str 62, 50937 Cologne
**4 Application of an equivalent project in another ethics committee**	Previous application and approval of this study; your number 15-407
**5 Written Consent by the director of the institute**	is available (see 10)
**6 Grants**	SFB TR-134, DZD-2.0 project area F, Doerenkamp-Stiftung
**7 Study-related extra costs**	none
**8 Advisory fees for industry sponsored studies**	none
**9 Multicenter study**	not applicable
**10 Declaration by the director of the institute**	„Hiermit erkläre ich gegenüber der Ethikkommission der Medizinischen Fakultät der Universität zu Köln, dass ich die sachlichen, personellen und organisatorischen Anforderungen der Studie gewährleiste."<br><br>Köln, den ....<br><br>Prof. Dr. med. J Brüning<br>(Director)

B. STUDIE

1 Scientific objective	Food is consumed in order to maintain energy balance at homeostatic levels. In addition, palatable food is also consumed for its hedonic properties independent of energy status. Such reward-related consumption can result in food intake exceeding homeostatic requirements and is considered a major cause in the rapidly increasing rates of obesity in developed countries. In comparison to homeostatic mechanisms of feeding, much less is known about how food intake is regulated by hedonic systems in the brain. Intriguingly, excessive consumption of palatable food can trigger neuroadaptive responses in brain reward circuitries as well as impulsive behavior similar to drugs of abuse (Kenny 2011).<br><br>Recently, different peptides (e.g. Insulin, Ghrelin, and Leptin) have been discussed to modulate both the hedonic experience of rewards and impulsive behavior; more recently, they were shown to directly affect the activity of dopamine (DA) neurons (Volkow, Wang et al. 2011).<br><br>Within this context, the peptide hormone Glucagon-like peptide 1 (Glp-1) has been identified as one of the main factors of the gut-brain axis to affect food intake (Gutzwiller, Goke et al. 1999, Wadden, Hollander et al. 2013). Its peripheral action leads to increased insulin secretion, which is the basis for its use in type 2 diabetes mellitus treatment (Ebert and Creutzfeldt 1987, Gutniak, Orskov et al. 1992). Its main effect in the central nervous system results in reduced food intake, which can only partly be explained by its actions on the homeostatic energy system in the hypothalamus (Ma, Bruning et al. 2007, Secher, Jelsing et al. 2014).<br><br>Thus, it is not surprising that recently Glp-1-receptors were also found to play a role in hedonic food intake, in particular in the context of reward learning (Richard, Anderberg et al. 2015). In a functional magnetic resonance imaging (fMRI) study, van Bloemendaal et al. (2015) demonstrated that administration of a Glp-1 analogue modulates neural activity in the brain reward circuitry during anticipation of reward in correlation to BMI.<br><br>Moreover, it has been shown (in animal studies) that Glp-1 suppresses mesolimbic dopamine signaling in the reward-receptive areas of the midbrain, in particular in the ventral tegmental area (VTA) and the nucleus accumbens (NAc). The expression of the dopaminergic auto-inhibitory receptor D2 was increased under Glp-1 in the VTA as well as the connectivity of DA neurons from the VTA to the medial shell of the NAc decreased (Mietlicki-Baase, Ortinski et al. 2013, Wang, Liu et al. 2015). Considering, that in the mesolimbic system dopamine encodes for reward learning by prediction error signaling (Eshel, Bukwich et al. 2015, Pignatelli and Bonci 2015), it is intriguing to think of Glp-1 as a possible

	mechanism causing behavioral alterations going along with food intake.<br><br>Here, we strive to scrutinize this modulatory role of Glp-1 on reward-related behavior in the context of food consumption. More specifically, we aim for relating neural activation, as revealed by fMRI in dopaminoreceptive areas in the midbrain as well as in mesolimbic and mesostriatal neural circuitry, to reward learning. Thereby, we simultaneously translate prior results gained from animal experiment to human behavior. Ultimately, our goal is a mechanistic understanding of the physiological effect of Glp-1 on human reward experience and effort learning.<br><br>In prior work, we (Sevgi et al. 2015) as well as others (e.g. Iglesias et al., 2014) have already established that prediction error activation from computational reward learning paradigms can robustly activate dopaminoreceptive midbrain (VTA/SN) and mesostriatal areas (NAc) in an fMRI paradigm. Data from animal experiments as well as human studies also revealed that obesity (or a High Fat Diet, HFD) leads to impaired prediction error signaling and altered reward learning from negative outcomes (Zhang, Manson et al. 2014, Sharpe, Clemens et al. 2015). Hence, it is not only of utmost interest if dopaminergic midbrain prediction error signaling is altered in obesity as a possible mechanism for overeating but also if this learning process is affected by Glp-1 receptor activation and can thus relate to a reducing effect on food intake by Glp-1 agonists.

2 Test plan	Methods: [ ] genotyping [x] behavioral studies [x] fMRI<br><br>The study is scheduled for a period of one year (January – December 2016).<br><br>The participants are recruited from a pre-existing database (Z-Project of the SFB TR 134, your Ethics approval No.: 10-226 and 12-020).<br><br>We are planning to include 20 lean control subjects and 20 obese subjects to investigate the modulation of the dopaminergic midbrain by Glp-1. We are planning to recruit participants for a behavioral study and a fMRI task focussing on effort spending in a food and monetary environment as well as reward learning, respectively.<br><br>All participants of this study will receive a Glp-1 (agonistic) analogue for one test day and a placebo for the other test day. The experiment will effectively span 1 week, including 2 test days:<br><br>(1) fMRI task and behavioral study with placebo<br>(2) fMRI task and behavioral study with Glp-1 analogue.<br><br>The order of the placebo and the Glp-1 analogue test day will be counter-balanced.<br><br>The evening before each test day, the participants are invited to the institute for a standardized dinner and the Glp-1/placebo injection. Prior to the Glp-1 analogue/placebo injection on the first evening, the study physician will briefly take the participant's medical history and, if applicable, perform an orientating check-up examination. On each test day, the participants will arrive at the institute at 8 am.<br><br>Subjects participating in the experiment will undergo the following procedures:<br><br>a) Blood measurement<br>Subjects will arrive fasted in the morning to the MRI laboratory of the Max-Planck-Institute for Metabolism Research.<br>On both test days, the study physician will take 4 tubes of venous blood (15ml) directly after arrival and 2 tubes (10ml) after the behavioral task to quantify metabolic parameters such as insulin, glucose (and consequently the HOMA-index), cholesterol (HDL and LDL), triglycerides, HbA1c and free fatty acids. The total volume of the blood draws will not exceed 25ml/test day.<br>Additionally, on both evenings before the test days, the blood glucose

	concentration will be measured using a blood droplet from a prick into the fingertip with a lancet.<br><br>b) Questionnaires<br>The participants will be asked to fill in the following online questionnaires before coming in for the first test:<br>- YFAS<br>- BES<br>- BDI-II<br>- FEV<br>- BIS-11<br>- WLOC<br>- PFS<br>- FGE.<br>Out of these, the BES (binge eating scale) and the BDI-II (Beck's depression inventor) will be used as exclusion criteria if positive.<br><br>c) fMRI<br>fMRI acquisition at baseline: A T1-weighted 3D MPRAGE sequence (8 minutes) will be performed to obtain a high-resolution structural image. Gradient-echo echo planar imaging will be used to measure the BOLD signal as an indication of cerebral activation in response to a probabilistic reward learning paradigm (Iglesias, Mathys et al. 2013) during a 40 minutes run.<br><br>Functional MR Imaging will be performed on a Siemens 3T Prisma scanner (Erlangen, Germany; maximum gradient strength 80 mT/m). Functional time series of each subject are acquired with a 64-channel head coil (Siemens, Erlangen, Germany). For functional time series, 42 axial slices (field of view 220 mm x 220 mm, 110x110 pixel matrix, thickness 2 mm, spacing 1 mm, resolution = 2x2x2mm3) parallel to the commissural line (AC-PC) will be acquired in a descending order from top to bottom using a single shot gradient echo-planar imaging sequence (EPI: TR = 2580 ms, TE = 26 ms, flip angle 90°).<br><br>d) Behavioral paradigm to assess willingness to work for food and monetary reward<br>All subjects will take part in this behavioral assessment on both test days. They will be asked to exert force on handles to measure their willingness to work for a presented food or monetary reward (Schmidt, d'Arc et al. 2008). This experiment will span 40 minutes. At the end of the experiment, the participant will receive one gained food reward and one gained monetary reward, which will be randomly assigned. The maximum possible rewards are up to 10 scoops of ice cream and 10

	Euros.<br><br>e) Glp-1 receptor activation via subcutaneous application of the Glp-1 analogue liraglutide (Victoza®)<br>To avoid a continuous subcutaneous infusion of recombinant GLP-1 (7-36) amide as a Glp-1 receptor agonist, the long-acting Glp-1 analogue liraglutide will be applied subcutaneously the evening before the test day. The lowest dose (0.6mg) will be used.<br><br>f) Placebo intervention<br>A Glp-1 placebo provided by NovoNordisc, which has been used in several studies, will be used (Ahmann, Rodbard et al. 2015).<br><br>g) Standardized dinner<br>As a standardized dinner participants will be served:<br>- 2 slices of bread<br>- 2 small slices of cheese, 2 small slices of salami<br>- butter (10g)<br>- 2 small tomatoes, 5 slices of cucumber<br>- 1 small fruit yoghurt<br>- 1 piece of fruit (e.g. one clementine)<br>- drinks: water and/or tea<br><br>h) Weight control<br>On each test day, the participants will be weighed on a mBCA (medical body composition analyser; seca mBCA 515, SECA GmbH, Hamburg, Germany) (Keane, Calton et al. 2015).<br><br>i) Medical history, medical examination<br>Prior to the injection of the Glp-1 analogue/ placebo on the first evening before the first test day, the study physician will briefly take the participant's medical history. This includes a short questionnaire about previous or known diseases or health problems of the participant. Furthermore, the physician will briefly ask the participant about any known diagnoses and, dependent on the disclosed health state, perform a short medical examination. This may include a body inspection, auscultation of the heart, lungs, arteries, percussion of the lungs, palpation of pulses and abdominal organs.<br><br>**Test Day 1: reward learning / effort for reward in lean and obese subjects**<br>Before participating on the first test day, subjects will give their informed consent and receive a copy of the signed document. Then, subjects will be asked to fill in the above mentioned online questionnaires.

	The evening before the first test day, participants will receive a standardized dinner as well as the placebo or Glp-1 analogue injection at the institute.<br>On the first test day, the participants will arrive fasted overnight at 7.45 h in the morning at the MRI Laboratory. After recording their weight with the mBCA scale, they will undergo the baseline blood draw, and then undergo the behavioral and the fMRI measurement.<br><br>*1. fMRI acquisition*<br>T1-weighted 3D MPRAGE sequence (8 minutes) will be performed to obtain a high-resolution anatomical image.<br>Echo planar imaging will be used to assess the BOLD signal in response to a reward learning paradigm during a 40 minutes run. A run consists of multiple blocks; appr. 120.<br><br>*2. Behavioral effort task*<br>In this task, the participant's willingness to spend effort for food and monetary rewards will be measured by assessing the force he/she spends on squeezing handles while being presented a possible reward (e.g. ice cream or money). At the end of the experiment, the participant will receive one gained food reward and one gained monetary reward, which will be randomly assigned. The maximum possible rewards are up to 10 scoops of ice cream and 10 Euros.<br><br>Please note that the order of the placebo and the Glp-1 analogue intervention is randomly assigned. For both measurements, both investigator and participant are blinded for the application of placebo or Glp-1 analogue.

	Overview of the randomly assigned groups<br>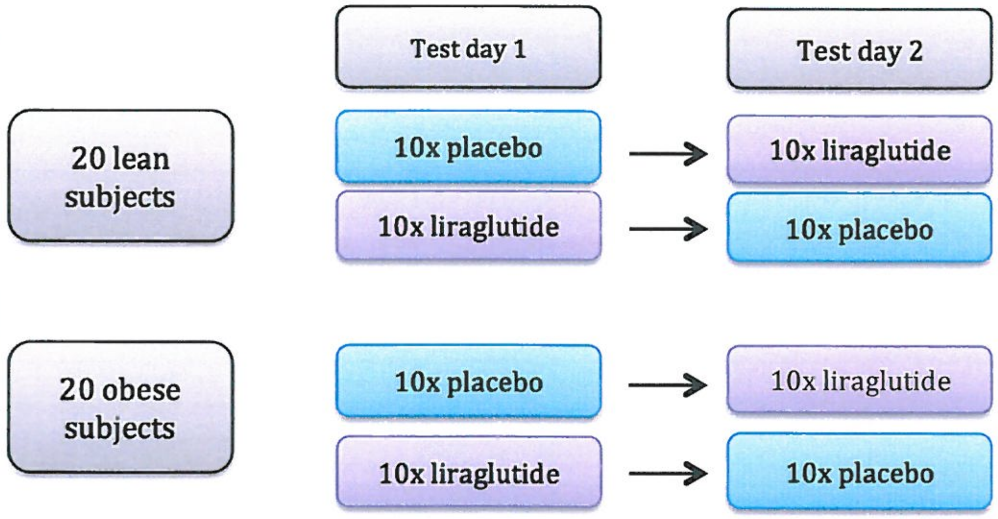<br><br>**Test Day 2: reward learning / effort for reward in lean and obese subjects**<br>The second test day equals the first test day. In short, after a standardized dinner and the placebo or Glp-1 analogue injection the night before the test day, participants will arrive fasted overnight at 7.45h at the PET/MRI laboratory of the MPI. After measuring the weight and the fasted blood draw the behavioral and the fMRI task will start.

	Timeline for the experiment<br>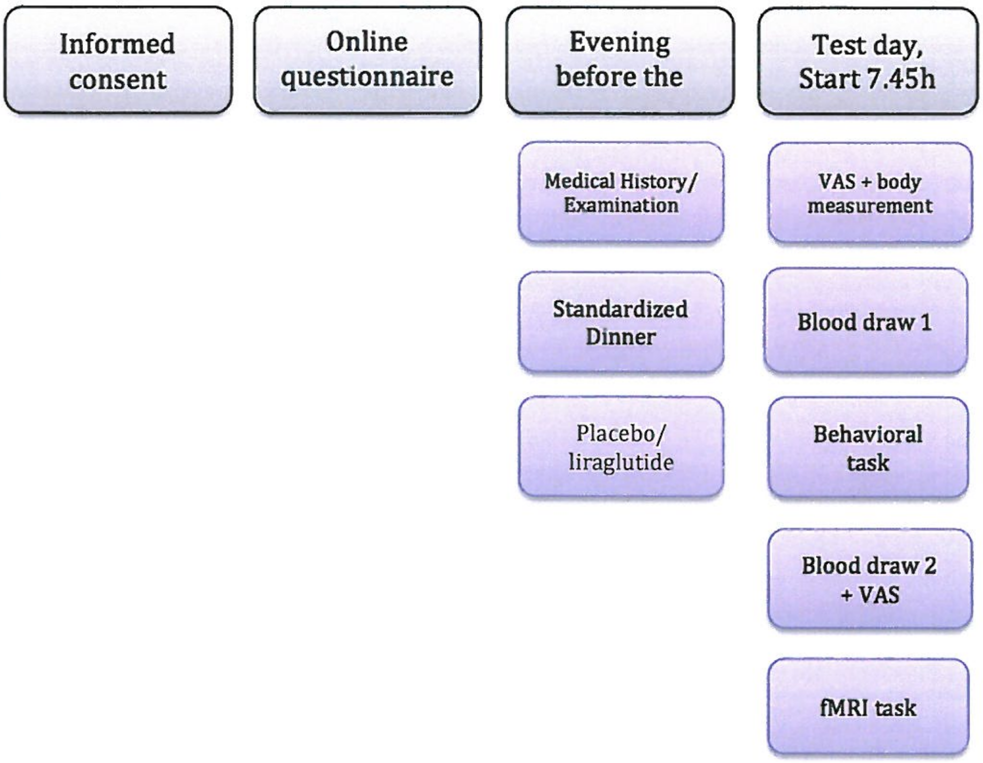
**3 Planned interventions and burden on subjects/liability**	The total duration of the imaging session is approximately 50 minutes and will be repeated with an interval of one week. Participants will need to be at the institute 4 times (2 evenings before the test days, 2 test days). The Glp-1 analogue will be injected by the study physician.
**4 Subjects**	Type of subjects: [x] Healthy [x] patients<br><br>Sample size: 40 participants. 20 lean subjects, 20 obese subjects<br><br>Inclusion criteria:<br><br>a) Informed consent obtained<br>b) Age: 20-40<br>c) Non-smoker (for the last 1 year not smoked more than 2 cigarettes per month)<br>d) Stable body weight (less than 5% self-reported change within the last 3 months)<br><br>Lean subjects with a BMI between 20-25 kg/m² (healthy

	weight range)<br><u>Obese subjects</u> with a BMI above 30 kg/m^2<br><br><u>Exclusion criteria</u>:<br>a) Serious or unstable medical illness (e.g., cancer)<br>b) Past or current history of alcoholism or consistent drug use<br>c) Current and history of major psychiatric illness as defined by the DSM-IV criteria including eating disorders<br>d) Medications that affect alertness (e.g., barbiturates, benzodiazepines, chloral hydrate, haloperidol, lithium, carbamazepine, phenytoin, etc.) and any psychoactive drugs or anti-obesity agents<br>e) History of major head trauma with loss of consciousness<br>f) Pregnancy<br>g) Nursing women<br>h) Kidney insufficiency (Kreatinin- Clearance <30 ml/min)<br>i) Reduced liver functioning<br>j) History of quincke edema<br>k) Family or personal history of multiple endocrine neoplasia type 2 (MEN2) or familial medullary thyroid carcinoma (FMTC)<br>l) Personal history of non-familial medullary thyroid carcinoma, other thyroid diseases<br>m) History of acute or chronic pancreatitis<br>n) Heart insufficiency NYHA I-IV<br>o) Chronic inflammatory bowel disease<br>p) Diabetic gastroparesis<br>q) Type I diabetes<br>r) Type 2 diabetes with oral antidiabetic treatment (in particular Sulfonylurea and basal insulin therapy)<br>s) Any known food allergy, certain food sensitivities (lactose); allergy towards active ingredient<br>t) History of metalworking, injury with shrapnel or metal slivers, and major surgery<br>u) History of pacemaker or neuro stimulator implantation<br>v) Dysphagia<br>w) Weight>150 kg<br><br>Subjects allowance: 80 Euros (after completion of the study)
**5 Type of trial**	These are behavioral studies and studies using functional and structural magnetic resonance imaging, which take place in the context of non-therapeutic biomedical research involving human subjects.
**6 Legal Regulation**	The data protection regulations will be respected. Compliance with these and the additional privacy policy of the Max Planck Society (MPG) are controlled by the data protection officer of the Max Planck Society.

**7 Unapproved drug**	not applicable
**8 Possible complications / risks**	Risks involved with MRI<br>When all MRI safety protocols are followed there is no known risk of MRI (Appendix A).<br><br>Risks associated with exposure to Liraglutide (Victoza®):<br>The European Medicines Agency has issued marketing authorisation for Liraglutide (Victoza®) on 30 June 2009 for treatment of type 2 diabetes mellitus up to a dose of 1.8 mg/day, in particular for patients failing to achieve optimal control on metformin alone or metformin + sulfonylurea therapy.<br>The most common side effects with Victoza® are nausea, vomiting, headache and diarrhoea. These side effects mainly vanish after the first few days. In this study, Victoza® is not used in combination with other anti-diabetic drugs so that no hypoglycemic events are expected.<br>Severe side effects like pancreatitis or any effects on thyroid malignomas are not expected due to the short study duration and the exclusion criteria. Quincke edema are seen very rarely with a probability of less than 1 to 1000 (but above 1 to 10 000 treated) and not expected in this study due to exclusion criteria.<br>Further prescribing information can be found in Appendix C.
**9 Benefit-to-risk assessment**	The risks of the study for participants in compliance with the exclusion criteria for MRI (Appendix A) and liraglutide intervention are minimal.<br>The study is intended exclusively for scientific progress and not meant to have any direct clinical impact.
**10 Interim analysis and termination criteria**	The collected data will be evaluated with internationally established standard programs for behavior analysis (SPSS, GraphPad PRISM) and for the analysis of imaging data (SPM and FSL) in accordance with the MPG guidelines for "good scientific practice". An interim analysis based on examination of half of the sample will be conducted. If there are any findings, which may adversely affect the risk-benefit ratio, the study may be terminated at the discretion of the program director.
**11 Content of the subjects informed consent statement**	See Appendix B
**12 Medical confidentiality and privacy agreement**	Medical confidentiality is guaranteed to full extent. All study participants will be assigned a pseudonym (4-digit numerical code) throughout the study. Privacy regulations are governed by the relevant guidelines of the Max Planck Society.

**13 Insurance cover**	The study will be assured by the general MPG police for testing subjects.
**14 Cover the costs of study**	Institute resources are available in sufficient amounts.

C.1 SUMMARY

Objective:
(1) The first aim of this study is to determine whether prediction errors in reward learning are altered in obesity and if Glp-1 receptor activation affects this learning process as a possible mechanism contributing to reduced food intake under Glp-1 analogues in normal weight and obesity.
(2) On a behavioral level, the second aim of this study is to determine whether Glp-1 receptor activation alters the motivation to spend effort for food or monetary rewards in normal weight and obesity.

Study design and type of examination:
This study is a randomized basic research study. We will conduct behavioral assessments with a computer based effort task as well as questionnaires. Further neuronal assessments will be conducted via a computational fMRI learning paradigm. We will examine BOLD (blood oxygen level dependent) activity changes in the VTA and ventral striatum/NAc after administration of the Glp-1 analogue Liraglutide or a placebo in lean controls and obese subjects.

Evaluation: The imaging data are analysed with SPM8 (functional imaging lab, University College London) in Matlab. The behavioral data will be analysed using SPSS or GraphPad Prism. Participants will remain anonymous in accordance with the "good scientific practice" guidelines of the Max Planck Society.

Ethical Issues: None

C.2 ZUSAMMENFASSUNG

Zielsetzung:
(1) In dieser Studie untersuchen wir zum einen, ob Vorhersagefehler in Belohnungslernprozessen bei Adipositas im Vergleich zu Normalgewicht verändert sind. Zum anderen untersuchen wir, ob eine Beeinflussung dieser Lernprozesse durch Glp-1 Rezeptoraktivierung in Adipösen und Normalgewichtigen ein möglicher Mechanismus für die reduzierte Nahrungsaufnahme unter Glp-1-Analoga sein kann.
(2) Auf der Verhaltensebene soll gezeigt werden, ob durch Glp-1 Rezeptoraktivierung in Adipösen und Normalgewichtigen die Motivation, für eine Belohnung körperliche Arbeit aufzuwenden, verändert werden kann.

Studienplanung und Art der Untersuchung:
Bei dieser Studie handelt es sich um eine randomisierte Grundlagenstudie.
Es werden Verhaltenstests (Fragebögen und eine Computeraufgabe zur Ermittlung der Motivation in Aussicht einer Belohnung) und neuronale Untersuchungen mittels computerbasierter funktioneller Magnetresonanztomographie (fMRT) mit einem Lernparadigma durchgeführt. Veränderungen der Gehirnaktivität im VTA und Ventralen Striatum/NAc werden anhand der Veränderungen der BOLD Aktivität nach einmaliger Gabe von Liraglutid bzw. Placebo bei schlanken Kontrollprobanden und Adipösen nachgewiesen.

Auswertung: Die Bildgebungsdaten werden mit SPM8 (functional imaging lab, University College London) in Matlab ausgewertet und die behavioralen Daten werden mittels SPSS oder GraphPad Prism analysiert. Die Probanden bleiben anonym entsprechend der Regeln zur "Good Scientific Practice" entsprechend den Bestimmungen der Max-Planck-Gesellschaft.

Ethische Belange berührende Problem: Keine.

Appendix

A Probandenaufklärung und Einverständniserklärung – fMRT-Untersuchung
B Probandenaufklärung und Einverständniserklärung – Glp-1 Studie
C Prescribing information (Fachinformation)

References

1. Ahmann, A., H. W. Rodbard, J. Rosenstock, J. T. Lahtela, L. de Loredo, K. Tornoe, A. Boopalan and M. A. Nauck (2015). "Efficacy and safety of liraglutide versus placebo added to basal insulin analogues (with or without metformin) in patients with type 2 diabetes: a randomized, placebo-controlled trial." Diabetes Obes Metab **17**(11): 1056-1064.
2. Ebert, R. and W. Creutzfeldt (1987). "Gastrointestinal peptides and insulin secretion." Diabetes Metab Rev **3**(1): 1-26.
3. Eshel, N., M. Bukwich, V. Rao, V. Hemmelder, J. Tian and N. Uchida (2015). "Arithmetic and local circuitry underlying dopamine prediction errors." Nature **525**(7568): 243-246.
4. Gutniak, M., C. Orskov, J. J. Holst, B. Ahren and S. Efendic (1992). "Antidiabetogenic effect of glucagon-like peptide-1 (7-36)amide in normal subjects and patients with diabetes mellitus." N Engl J Med **326**(20): 1316-1322.
5. Gutzwiller, J. P., B. Goke, J. Drewe, P. Hildebrand, S. Ketterer, D. Handschin, R. Winterhalder, D. Conen and C. Beglinger (1999). "Glucagon-like peptide-1: a potent regulator of food intake in humans." Gut **44**(1): 81-86.
6. Iglesias, S., C. Mathys, K. H. Brodersen, L. Kasper, M. Piccirelli, H. E. den Ouden and K. E. Stephan (2013). "Hierarchical prediction errors in midbrain and basal forebrain during sensory learning." Neuron **80**(2): 519-530.
7. Keane, K. N., E. K. Calton, V. F. Cruzat, M. J. Soares and P. Newsholme (2015). "The impact of cryopreservation on human peripheral blood leucocyte bioenergetics." Clin Sci (Lond) **128**(10): 723-733.
8. Kenny, P. J. (2011). "Reward mechanisms in obesity: new insights and future directions." Neuron **69**(4): 664-679.
9. Ma, X., J. Bruning and F. M. Ashcroft (2007). "Glucagon-like peptide 1 stimulates hypothalamic proopiomelanocortin neurons." J Neurosci **27**(27): 7125-7129.
10. Mietlicki-Baase, E. G., P. I. Ortinski, L. E. Rupprecht, D. R. Olivos, A. L. Alhadeff, R. C. Pierce and M. R. Hayes (2013). "The food intake-suppressive effects of glucagon-like peptide-1 receptor signaling in the ventral tegmental area are mediated by AMPA/kainate receptors." Am J Physiol Endocrinol Metab **305**(11): E1367-1374.
11. Pignatelli, M. and A. Bonci (2015). "Role of Dopamine Neurons in Reward and Aversion: A Synaptic Plasticity Perspective." Neuron **86**(5): 1145-1157.
12. Richard, J. E., R. H. Anderberg, A. Goteson, F. M. Gribble, F. Reimann and K. P. Skibicka (2015). "Activation of the GLP-1 receptors in the nucleus of the solitary tract reduces food reward behavior and targets the mesolimbic system." PLoS One **10**(3): e0119034.
13. Schmidt, L., B. F. d'Arc, G. Lafargue, D. Galanaud, V. Czernecki, D. Grabli, M. Schupbach, A. Hartmann, R. Levy, B. Dubois and M. Pessiglione (2008). "Disconnecting force from money: effects of basal ganglia damage on incentive motivation." Brain **131**(Pt 5): 1303-1310.
14. Secher, A., J. Jelsing, A. F. Baquero, J. Hecksher-Sorensen, M. A. Cowley, L. S. Dalboge, G. Hansen, K. L. Grove, C. Pyke, K. Raun, L. Schaffer, M. Tang-Christensen, S. Verma, B. M. Witgen, N. Vrang and L. Bjerre Knudsen (2014). "The arcuate nucleus mediates GLP-1 receptor agonist liraglutide-dependent weight loss." J Clin Invest **124**(10): 4473-4488.

15. Sharpe, M. J., K. J. Clemens, M. J. Morris and R. F. Westbrook (2015). "Daily Exposure to Sucrose Impairs Subsequent Learning About Food Cues: A Role for Alterations in Ghrelin Signaling and Dopamine D2 Receptors." Neuropsychopharmacology.
16. Volkow, N. D., G. J. Wang and R. D. Baler (2011). "Reward, dopamine and the control of food intake: implications for obesity." Trends Cogn Sci **15**(1): 37-46.
17. Wadden, T. A., P. Hollander, S. Klein, K. Niswender, V. Woo, P. M. Hale and L. Aronne (2013). "Weight maintenance and additional weight loss with liraglutide after low-calorie-diet-induced weight loss: the SCALE Maintenance randomized study." Int J Obes (Lond) **37**(11): 1443-1451.
18. Wang, X. F., J. J. Liu, J. Xia, J. Liu, V. Mirabella and Z. P. Pang (2015). "Endogenous Glucagon-like Peptide-1 Suppresses High-Fat Food Intake by Reducing Synaptic Drive onto Mesolimbic Dopamine Neurons." Cell Rep **12**(5): 726-733.
19. Zhang, Z., K. F. Manson, D. Schiller and I. Levy (2014). "Impaired associative learning with food rewards in obese women." Curr Biol **24**(15): 1731-1736.
