# Supplementary material for: Liraglutide restores impaired associative learning in individuals with obesity
Source: Nat Metab. 2023 Aug 17;5(8):1352–63. doi: 10.1038/s42255-023-00859-y (PMC10447249; doi:10.1038/s42255-023-00859-y)
Supplement: Supplementary file 2 — Reporting Summary [file 42255_2023_859_MOESM2_ESM.pdf]

nature portfolio

Corresponding author(s): Marc Tittgemeyer

Last updated by author(s): Jun 15, 2023

Reporting Summary

Nature Portfolio wishes to improve the reproducibility of the work that we publish. This form provides structure for consistency and transparency in reporting. For further information on Nature Portfolio policies, see our Editorial Policies and the Editorial Policy Checklist.

Statistics

For all statistical analyses, confirm that the following items are present in the figure legend, table legend, main text, or Methods section.

n/a	Confirmed	
☐	☒	The exact sample size (n) for each experimental group/condition, given as a discrete number and unit of measurement
☐	☒	A statement on whether measurements were taken from distinct samples or whether the same sample was measured repeatedly
☐	☒	The statistical test(s) used AND whether they are one- or two-sided<br>*Only common tests should be described solely by name; describe more complex techniques in the Methods section.*
☐	☒	A description of all covariates tested
☐	☒	A description of any assumptions or corrections, such as tests of normality and adjustment for multiple comparisons
☐	☒	A full description of the statistical parameters including central tendency (e.g. means) or other basic estimates (e.g. regression coefficient) AND variation (e.g. standard deviation) or associated estimates of uncertainty (e.g. confidence intervals)
☐	☒	For null hypothesis testing, the test statistic (e.g. F, t, r) with confidence intervals, effect sizes, degrees of freedom and P value noted<br>*Give P values as exact values whenever suitable.*
☒	☐	For Bayesian analysis, information on the choice of priors and Markov chain Monte Carlo settings
☐	☒	For hierarchical and complex designs, identification of the appropriate level for tests and full reporting of outcomes
☐	☒	Estimates of effect sizes (e.g. Cohen's d, Pearson's r), indicating how they were calculated

Our web collection on statistics for biologists contains articles on many of the points above.

Software and code

Policy information about availability of computer code

Data collection	MATLAB R2019b (The Mathworks Inc., Massachusetts, USA), Cogent2000 (http://www.vislab.ucl.ac.uk/ Cogent/index.html)
Data analysis	Statistical Parametric Mapping (SPM), version 12 (r6225, Wellcome Trust Centre for Neuroimaging, London, UK) implemented in MATLAB R2019b (The Mathworks Inc., Massachusetts, USA), TAPAS toolbox (vers. 1.0; http://www.translationalneuromodeling.org/tapas), RStudio (vers. 1.4.1717) and R (vers. 4.0.0)<br>R packages: 'lme4' R-package (vers. 1.1-26), 'lmerTest' R-package (vers. 3.1-3), Wilcox' WRS functions (vers. 1.1-0)<br>G*Power (version 3.1)<br>FMRIB Software Library (FSL version 5.08, http://www.fmrib.ox.ac.uk/fsl)<br>FSL tools: automated brain extraction tool, FSL's MCFLIRT, TOPUP, ICA-based X-noiseifier

For manuscripts utilizing custom algorithms or software that are central to the research but not yet described in published literature, software must be made available to editors and reviewers. We strongly encourage code deposition in a community repository (e.g. GitHub). See the Nature Portfolio guidelines for submitting code & software for further information.

Data

Policy information about availability of data

All manuscripts must include a data availability statement. This statement should provide the following information, where applicable:

- Accession codes, unique identifiers, or web links for publicly available datasets
- A description of any restrictions on data availability
- For clinical datasets or third party data, please ensure that the statement adheres to our policy

The human data reported in this study cannot be deposited in a public repository per GDPR and IRB data protection policies. To request access, please contact [Marc Tittgemeyer, Max Planck Institute for Metabolism Research, tittgemeyer@sf.mpg.de]. Data provision may include processed and unprocessed data and will require a data-sharing agreement. Data sharing necessitates that the purpose of data re-analysis is in line with the study aims as approved by the ethics review boards and participants consent. Furthermore, consent to data privacy need to be assured by signing the agreement from accordingly. Requests will be answered within 4 weeks.

Research involving human participants, their data, or biological material

Policy information about studies with human participants or human data. See also policy information about sex, gender (identity/presentation), and sexual orientation and race, ethnicity and racism.

Reporting on sex and gender	Sex was determined based on self-reporting. Data on gender were not collected. Neither sex nor gender were included in our analysis as there are no hypotheses driven studies suggesting sex-dimorphism relating to insulin-receptor signalling or GLP-1-receptor signaling (in dopamine neuron) -neither at the starting point of the study (2016) nor now.
Reporting on race, ethnicity, or other socially relevant groupings	no socially relevant cathegorizaion was made in the selection of study participants
Population characteristics	Age 26y; BMI (insulin sensitive group): 22.27 +/- 0.27 kg/m2; BMI (insulin resistant group): 36.23 +/- 0.74 kg/m2; blood insulin levels (insulin sensitive group): 6 +/- 0.5 mU/l; blood insulin levels (insulin resistant group): 14 +/- 1.4 mU/l; blood glucose levels (insulin sensitive group): 81 mg/dl; blood glucose levels (insulin resistant group): 86 mg/dl;
Recruitment	All participants were recruited from the pre-existing database of volunteers maintained at the Max Planck Institute for Metabolism Research. Registered subjects received an online invitation for study participation. We specifically invited participants with a healthy BMI (<25kg/m2) or with obesity (BMI>= 29kg/m2) to ensure recruitment of participants with normal and impaired insulin sensitivity. Even though BMI measurements in our database rely in self-reported weight and height, BMI was controlled on both measurement days to minimise self-reporting bias.
Ethics oversight	Ethics committee of the Medical Faculty of the University of Cologne (No. 16-251)

Note that full information on the approval of the study protocol must also be provided in the manuscript.

Field-specific reporting

Please select the one below that is the best fit for your research. If you are not sure, read the appropriate sections before making your selection.

☒ Life sciences ☐ Behavioural & social sciences ☐ Ecological, evolutionary & environmental sciences

For a reference copy of the document with all sections, see nature.com/documents/nr-reporting-summary-flat.pdf

Life sciences study design

All studies must disclose on these points even when the disclosure is negative.

Sample size	An a-priori power analysis was performed in G*power based on an estimated effect size. Expecting a drop-out rate of 25%, fifty-four healthy volunteers were recruited, based on a power analysis assuming an alpha (significance) value of 0.05, a power of 0.95 and a medium effect size relating to Cohen's d = 0.6. The power estimation (G*Power Version 3.1) was performed assuming a mixed-effect model with repeated measurements in two groups (lean vs obese) and considering within and between-group interaction (liraglutide vs placebo), yielding a total sample size of N = 40.
Data exclusions	Individual sessions were excluded from data analysis based on elimination criteria regarding task performance and excessive head motion to avoid artefacts in fMRI data. In total, 40 sessions (20 placebo session and 20 GLP-1 sessions) were excluded due to the following reasons: 5 sessions with more than 20% invalid trials (missing response or a response later than 1.5 s), 17 sessions with less than 65% accuracy, 6 sessions with subjects always pressing the same button, 1 session due to technical problems, 1 session because the computational model could not be fitted, and 10 sessions with excessive head motion (maximal framewise displacement > 4mm). In consequence, a total of 68 individual sessions (34 placebo session and 34 GLP-1 sessions) from 43 different subjects, 23 lean and 20 obese, were included into the analysis (see Table 1 for sample characteristics). The final sample (N = 43 with 68 included sessions) allowed for a power of 0.62 for the endpoint of our model analysis (adaptive prediction error) given a two-way interaction of insulin sensitivity (normal vs. impaired) × intervention (GLP-1 vs. placebo) within the used mixed effect models; relating to an effect sizes of Cohen's f = 0.1 and 0.15, respectively, at

	the significance level alpha = 0.05.
Replication	All processing scripts have been commented and uploaded to a version controlled repository (local git server). All data have been stored in a database system subject to a auditing trail and revision history. Data, experimental procedure protocols and analysis code can be made available for reproduction/replication (see above).
Randomization	Participants were randomly assigned to the order of the intervention (placebo or GLP-1)
Blinding	While the participants were blinded to the order of the intervention, the investigators were not to ensure participant safety on the highest level possible (e.g. in case of allergic reaction). As the study physician also performed the data analysis, blinding could not be ensure for analyses.

Reporting for specific materials, systems and methods

We require information from authors about some types of materials, experimental systems and methods used in many studies. Here, indicate whether each material, system or method listed is relevant to your study. If you are not sure if a list item applies to your research, read the appropriate section before selecting a response.

Materials & experimental systems

n/a	Involved in the study	
☒	☐	Antibodies
☒	☐	Eukaryotic cell lines
☒	☐	Palaeontology and archaeology
☒	☐	Animals and other organisms
☐	☒	Clinical data
☒	☐	Dual use research of concern
☒	☐	Plants

Methods

n/a	Involved in the study	
☒	☐	ChIP-seq
☒	☐	Flow cytometry
☐	☒	MRI-based neuroimaging

Clinical data

Policy information about clinical studies

All manuscripts should comply with the ICMJE guidelines for publication of clinical research and a completed CONSORT checklist must be included with all submissions.

Clinical trial registration	The study is not a clinical trial, but a basic research study involving humans.
Study protocol	The protocol based on which this study was approved by the IRB is part of the Supplementary Material
Data collection	All participants were recruited from the pre-existing database of volunteers maintained at the Max Planck Institute for Metabolism Research in Cologne, Germany. Participants were invited according to BMI (<25 kg/m2 or >=30 kg/m2) to ensure collection of participants with high and low insulin sensitivity. Participants were required to have no known diseases. Recruitment and data collection were performed between 30.06.2016 and 24.08.2017.
Outcomes	primary endpoints: adaptive learning, prediction error encoding and cerebral neuronal activity. As secondary outcomes, plasma insulin and glucose, HOMA-IR, and hunger scores.

Magnetic resonance imaging

Experimental design

Design type	task-based (sensory-sensory learning); event-related
Design specifications	Each participant undertook the learning task twice; each session consisted of 320 trials divided into 10 blocks. Block length (24-40 trials) and block sequence were varied randomly across blocks. Cues were presented for 300 ms, response interval comprised 1200 ms, the duration of visual outcome presentations was 300 ms. Inter-trial interval (ITI) varied randomly between 1.5 and 2.5 s.
Behavioral performance measures	We modeled the trial-by-trial changes in participant's choices with the Hierarchical Gaussian Filter yielding subject-specific parameter estimates and learning trajectories. Participants' task performance was tested according to their accuracy rate. Only participants with >65% correct answers were included in the analysis.

Acquisition

Imaging type(s)	functional MRI
Field strength	3T
Sequence & imaging parameters	fMRI data were acquired in one session with a T2-weighted (gradient-echo) echo-planar imaging sequence (31 axial slices, slice thickness: 2 mm; in-plane resolution: 2 mm × 2 mm; no distance factor; ascending interleaved in-plane acquisition; TR = 2000 ms; TE = 30 ms; flip angle = 90°; field of view = 224 × 224 × 60 mm^3).
Area of acquisition	whole-brain

Diffusion MRI ☐ Used ☒ Not used

Preprocessing

Preprocessing software	FMRIB Software Library (FSL version 5.08), FSL's automated brain extraction tool, realignment:FSL's MCFLIRT, distortion correction: TOPUP; smoothing: 8 mm FWHM Gaussian kernel;
Normalization	functional data were co-registered to the subject's T1-weighted image and normalised to the MNI standard space
Normalization template	ICBM152
Noise and artifact removal	Structured artifacts were removed using independent component analysis followed by FSL's ICA-based X-noiseifier.
Volume censoring	24 motion parameters —six parameters relating to the current and the preceding volume, respectively, plus each of these matrices squared— mean signal extracted from the ventricular cerebrospinal fluid, and a matrix with motion-outlier volumes –identified using the tool fsl_motion_outliers, dvars option targeting global intensity differences between subsequent volumes, at a threshold of 75th percentile + 2.5 * interquartile range. The maximum framewise displacement (maxFD) as a measure of motion between slices did not differ between groups and interventions. Low-frequency signal drifts were filtered using a cut-off of 128 s.

Statistical modeling & inference

Model type and settings	GLM on 1st and 2nd level; 1st: conditions were modeled using a boxcar reference vector convolved with the canonical hemodynamic response function and its time derivative; BOLD response to outcomes was parametrically modulated by the task derived variable, 2nd: flexible factorial design.
Effect(s) tested	In a flexible factorial design, the factors subject, group, and intervention were specified, with all variances set to unequal and dependency set to 1 for intervention, otherwise to 0 assuming unequal variance for the factor subject, making the inclusion of random subject blocks unnecessary. The GLM included four regressors.

Specify type of analysis: ☒ Whole brain ☐ ROI-based ☐ Both

Statistic type for inference (See Eklund et al. 2016)	cluster-wise, see below
Correction	Group-level results were thresholded at $p < .05$, FWE-corrected at cluster level, with a cluster-defining threshold of $p < .001$

Models & analysis

n/a	Involved in the study
☒	☐ Functional and/or effective connectivity
☒	☐ Graph analysis
☒	☐ Multivariate modeling or predictive analysis
