# Supplementary material for: Liraglutide restores impaired associative learning in individuals with obesity
Source: Nat Metab. 2023 Aug 17;5(8):1352–63. doi: 10.1038/s42255-023-00859-y (PMC10447249; doi:10.1038/s42255-023-00859-y)
Supplement: Supplementary file 3 — Test for the effects of intervention (placebo and liraglutide) and group (IS+ and IS–) on learning parameters, including post hoc tests. [file 42255_2023_859_MOESM3_ESM.pdf]

Supplemental Table 1. Learning model parameters

A: Sensory Prediction Error - effect of group (IS+ vs. IS–) on the sensory prediction error under placebo conditions

	df	*t*	*p*		Cohen's *d*
group	332.82	-0.0085	0.9932		-9.35e-04

Note. Welch two-sided two-sample t-test, df = degrees of freedom

B: Adaptive Learning Rate - effect of group (IS+ vs IS–) on the adaptive learning rate under placebo conditions

	df	*t*	*p*		Cohen's *d*
group	98.842	2.2425	0.02716	*	0.44

Note. Welch two-sided two-sample t-test, df = degrees of freedom

C: Adaptive Prediction Error - effect of group (IS+ vs IS–) on the adaptive prediction error under placebo conditions

	df	*t*	*p*		Cohen's *d*
group	321.03	2.5003	0.01291	*	0.28

Note. Welch two-sided two-sample t-test, df = degrees of freedom

D: Sensory Prediction Error – no significant effect of group (IS+ vs IS–) or intervention (placebo vs liragludide) on sensory prediction error detectable (Model M1)

	Sum Sq	Mean Sq	NumDF	DenDF	*F*	*p*		Cohen's *f* (partial)
group	0.0052	0.0052	1	656	1.184	0.277		0.04
int	0.0052	0.0052	1	656	1.1991	0.274		0.04
pred	10.5758	10.5758	1	656	2424.2537	< 0.001	***	1.92
correct	0.0384	0.0384	1	656	8.8034	0.003	**	0.12
group:int	0.0004	0.0004	1	656	0.0952	0.758		0.01
group:pred	0.0048	0.0048	1	656	1.0996	0.295		0.04
int:pred	0.002	0.002	1	656	0.4518	0.502		0.03
group:correct	0.0001	0.0001	1	656	0.0139	0.906		4.61e-03
int:correct	0.0001	0.0001	1	656	0.0313	0.860		6.91e-03
pred:correct	0.212	0.212	1	656	48.6045	< 0.001	***	0.27
group:int:pred	0	0	1	656	0	0.998		7.12e-05
group:int:correct	0.0051	0.0051	1	656	1.1586	0.282		0.04
group:pred:correct	0.0002	0.0002	1	656	0.0411	0.839		7.92e-03
int:pred:correct	0.002	0.002	1	656	0.453	0.501		0.03
group:int:pred:correct	0.0071	0.0071	1	656	1.6162	0.204		0.05

Note. Type III Analysis of Variance Table with Satterthwaite's method based on a mixed effect model; NumDF = degrees of freedom in the numerator, DenDF = degrees of freedom in the denominator, int = intervention; pred = predictability of the trial, correct = correctness of a subject's choice

E: Learning rate - effect of intervention (placebo vs GLP-1) on the learning rate depends on group (IS+ vs IS–; Model M2)

	Sum Sq	Mean Sq	NumDF	DenDF	*F*	*p*		Cohen's *f* (partial)
group	0.155	0.155	1	57.8	1.0933	0.300		0.14
int	0.0739	0.0739	1	626.22	0.5211	0.471		0.03
pred	13.1702	13.1702	1	622.92	92.9226	< 0.001	***	0.39
group:int	2.0541	2.0541	1	626.22	14.4928	< 0.001	***	0.15
Posthoc Analysis of group x intervention interaction								
	estimate	SE	*df*		*t*	*p*		
IS+ Placebo - IS– Placebo	0.3732	0.1736	44		2.149	0.154		
IS+ Placebo - IS+ GLP-1	0.1499	0.0466	640		3.215	0.007	**	
IS+ Placebo - IS– GLP-1	0.0587	0.174	44.3		0.337	0.987		
IS– Placebo - IS+ GLP-1	-0.2233	0.1733	43.7		-1.288	0.575		
IS– Placebo - IS– GLP-1	-0.3145	0.0485	641.2		-6.481	< 0.001	***	
IS+ GLP-1 - IS– GLP-1	-0.0912	0.1737	44		-0.525	0.952		
group:pred	0.0731	0.0731	1	622.92	0.5154	0.473		0.03
int:pred	0.0138	0.0138	1	622.92	0.0973	0.755		0.01
group:int:pred	0.1602	0.1602	1	622.92	1.1303	0.288		0.04

Note. Type III Analysis of Variance Table with Satterthwaite's method based on a mixed effect model; Posthoc-analysis was calculated using the Tukey's procedure; NumDF = degrees of freedom in the numerator, DenDF = degrees of freedom in the denominator, int = intervention; pred = predictability of the trial, correct = correctness of a subject's choice, SE = standard error, *df* = degrees of freedom.

F: Adaptive Prediction Error – effect of intervention (placebo vs liraglutide) on the adaptive prediction error depends on group (IS+ vs IS–; Model M3)

	Sum Sq	Mean Sq	NumDF	DenDF	*F*	*p*		Cohen's f (partial)
group	0.384	0.384	1	121.51	3.0184	0.085		0.16
int	0.206	0.206	1	620.23	1.6183	0.204		0.05
pred	40.425	40.425	1	614.69	318.0383	< 0.001	***	0.72
correct	0.836	0.836	1	614.95	6.5789	0.010	*	0.10
group:int	0.733	0.733	1	620.23	5.7658	0.017	*	0.10
**Posthoc Analysis of group : intervention interaction**								
	**estimate**	**SE**		**df**	***t***	***p***		
IS+ Placebo - IS– Placebo	0.2143	0.1037		48.6	2.066	0.179		
IS+ Placebo - IS+ GLP-1	0.0915	0.0436		649.1	2.098	0.155		
IS+ Placebo - IS– GLP-1	0.0318	0.1043		49.5	0.305	0.990		
IS– Placebo - IS+ GLP-1	-0.1228	0.1033		47.8	-1.189	0.637		
IS– Placebo - IS– GLP-1	-0.1826	0.0454		650.8	-4.025	< 0.001	***	
IS+ GLP-1 - IS– GLP-1	-0.0598	0.1038		48.8	-0.576	0.939		
group:pred	0.269	0.269	1	614.69	2.1132	0.146		0.06
int:pred	0.054	0.054	1	614.69	0.4221	0.516		0.03
group:correct	0.06	0.06	1	614.95	0.4687	0.494		0.03
int:correct	0.047	0.047	1	614.96	0.3714	0.542		0.02
pred:correct	0.029	0.029	1	614.87	0.2278	0.633		0.02
group:int:pred	0.107	0.107	1	614.69	0.8409	0.359		0.04
group:int:correct	0.01	0.01	1	614.96	0.0749	0.784		0.01
group:pred:correct	0.007	0.007	1	614.87	0.0571	0.811		9.63e-03
int:pred:correct	0.018	0.018	1	614.88	0.1392	0.709		0.02
group:int:pred:correct	0.024	0.024	1	614.88	0.1914	0.661		0.02

Note. Type III Analysis of Variance Table with Satterthwaite's method based on a mixed effect model; Posthoc-analysis was calculated using the Tukey's procedure; NumDF = degrees of freedom in the numerator, DenDF = degrees of freedom in the denominator, int = intervention; pred = predictability of the trial, correct = correctness of a subject's choice, SE = standard error, df = degrees of freedom.
