# Supplementary material for: Liraglutide restores impaired associative learning in individuals with obesity
Source: Nat Metab. 2023 Aug 17;5(8):1352–63. doi: 10.1038/s42255-023-00859-y (PMC10447249; doi:10.1038/s42255-023-00859-y)
Supplement: Supplementary file 4 — Neural tracking of the adaptive prediction error. [file 42255_2023_859_MOESM4_ESM.pdf]

Supplementary Table S2. Neural tracking of the adaptive prediction error

	Cluster level		Peak level			
	$p_{FWE\text{-}corr}$	**Size**	***t***	**x**	**y**	**z**
**Conjunction**						
Putamen	> 0.001	2740	5.93	32	-10	4
NAc			5.13	8	10	-10
mid Insula			4.58	-40	0	8
vmPFC	> 0.001	1371	4.32	8	34	-2
vmPFC			3.96	6	44	-8

Note. Statistics for *T*-contrasts identifying brain regions, which encode the adaptive prediction error. A conjunction analysis of the four regressors IS+placebo, IS+GLP-1, IS–placebo, and IS–GLP-1 was applied. Statistical threshold was $p < .05$, FWE-corrected at cluster level, with underlying voxel-level threshold of $p < 0.001$. vmPFC = ventromedial prefrontal cortex; NAc = nucleus accumbens.
