# Supplementary material for: Liraglutide restores impaired associative learning in individuals with obesity
Source: Nat Metab. 2023 Aug 17;5(8):1352–63. doi: 10.1038/s42255-023-00859-y (PMC10447249; doi:10.1038/s42255-023-00859-y)
Supplement: Supplementary file 5 — Standardized dinner the evening before the testing day. [file 42255_2023_859_MOESM5_ESM.pdf]

Supplementary Table S3. Standardized dinner the evening prior to the testing day**.**

Item	Amount	kcal	Fat(g)	CH(g)	Protein (g)
Bread	2 slices , 50 g each	241	2.7	45	7.5
Cheese	Slices à 17g	321	24	0.3	26
Salami	Slices à 18g	306	25	1	19
Butter	16.7 g	743	82	0.6	0.7
Tomato	2 small tomatoes				
Cucumber	5 slices				
Fruit Yoghurt	1 yoghurt à 150g	143	4.2	21.8	4.5
Fruit	1 small Apple	55	0.60	11	0.3
Drinks	Water at free disposal				

Note. Kcal, fat, CH, protein content per 100g are displayed, CH = Carbohydrates
